# A chromosome level reference genome of Diviner’s sage (*Salvia divinorum*) provides insight into salvinorin A biosynthesis

**DOI:** 10.1186/s12870-024-05633-0

**Published:** 2024-10-01

**Authors:** Scott A. Ford, Rob W. Ness, Moonhyuk Kwon, Dae-Kyun Ro, Michael A. Phillips

**Affiliations:** 1 Department of Cell and Systems Biology, University of Toronto, Toronto, ON M5S 3G5 Canada; 2 Department of Biology, University of Toronto - Mississauga, Mississauga, ON L5L 1C6 Canada; 3 Department of Ecology and Evolutionary Biology, University of Toronto, Toronto, ON M5S 3G5 Canada; 4 Department of Biological Sciences, University of Calgary, Calgary, AB T2N 1N4 Canada; 5 Present Address: Division of Applied Life Science (BK21 Four), ABC-RLRC, RIMA, Gyeongsang National University, Jinju, 52828 Republic of Korea

**Keywords:** *Salvia divinorum*, Diterpenoid biosynthesis, De novo genome assembly, Lamiaceae, Neoclerodane diterpenes, Κ opioid receptor, Medicinal plants

## Abstract

**Background:**

Diviner’s sage (*Salvia divinorum;* Lamiaceae) is the source of the powerful hallucinogen salvinorin A (SalA). This neoclerodane diterpenoid is an agonist of the human *Κ*-opioid receptor with potential medical applications in the treatment of chronic pain, addiction, and post-traumatic stress disorder. Only two steps of the approximately twelve step biosynthetic sequence leading to SalA have been resolved to date.

**Results:**

To facilitate pathway elucidation in this ethnomedicinal plant species, here we report a chromosome level genome assembly. A high-quality genome sequence was assembled with an N50 value of 41.4 Mb and a BUSCO completeness score of 98.4%. The diploid (2n = 22) genome of ~ 541 Mb is comparable in size and ploidy to most other members of this genus. Two diterpene biosynthetic gene clusters were identified and are highly enriched in previously unidentified cytochrome P450s as well as crotonolide G synthase, which forms the dihydrofuran ring early in the SalA pathway. Coding sequences for other enzyme classes with likely involvement in downstream steps of the SalA pathway (BAHD acyl transferases, alcohol dehydrogenases, and O-methyl transferases) were scattered throughout the genome with no clear indication of clustering. Differential gene expression analysis suggests that most of these genes are not inducible by methyl jasmonate treatment.

**Conclusions:**

This genome sequence and associated gene annotation are among the highest resolution in *Salvia*, a genus well known for the medicinal properties of its members. Here we have identified the cohort of genes responsible for the remaining steps in the SalA pathway. This genome sequence and associated candidate genes will facilitate the elucidation of SalA biosynthesis and enable an exploration of its full clinical potential.

**Supplementary Information:**

The online version contains supplementary material available at 10.1186/s12870-024-05633-0.

## Introduction

*Salvia divinorum* (Epling and Játiva-M.), also known as Ska María Pastora or Diviner’s sage, is a hallucinogenic plant used in ritualistic ceremonies by the Mazatec people of Oaxaca, Mexico [1]. Its hallucinogenic properties are due to salvinorin A (SalA), a neoclerodane diterpenoid produced in glandular trichomes on the surfaces of leaves. SalA is a highly selective and potent *K*-opioid receptor (KOR) agonist and the first known nitrogen-free ligand for this receptor family [2]. Its powerful dissociative properties and popularity as a recreational drug have led to its criminalization in Canada, much of western Europe, Japan, South Korea, and 33 states of the United States. However, it remains legal in many countries, including Mexico, home to its only natural endemic population.

SalA belongs to the neoclerodane subfamily of labdane diterpenoid secondary metabolites. Diterpenoids represent enormous chemical diversity in the plant kingdom numbering close to 13,000, including the salvinorins unique to *S. divinorum*. [3–5]. Although widespread in the Viridiplantae, diterpenoids are especially abundant in the Lamiaceae, which are the source of approximately one quarter (~ 3,000) of this total.

There are at least 22 diterpenes in the neoclerodane diterpenoid group that have now been isolated from *S. divinorum* [6], but SalA is the most abundant and the only natural product in this species thus far to demonstrate pharmacological activity [7]. It is the first reported diterpene to possess psychoactive properties [8]. It displays high affinity for the KOR (K_i_ of ~ 4 nM), one of five related opioid receptors in the human body that modulate pain, emotional control, memory, learning, and mood (Schwarzer 2009). It has thus been at the center of research efforts to understand the medical potential of this plant species. The selectivity and potency of SalA towards KOR suggest therapeutic potential in the treatment of pain, inflammation, and depression [9–11]. Pain relief has traditionally relied on agonism of the related μ opioid receptor (MOR), which leads to dependency and addiction. In contrast, agonism of KOR is not associated with addiction and therefore represents a critical alternative in the development of pharmaceuticals to treat chronic pain. Indeed, SalA agonism of KOR may exert anti-addictive effects [7, 12].

Other clerodane diterpenoids of *S. divinorum* such as salvinorin B and divinatorin D and E are also KOR agonists with K_i_ values ranging from 65 to 418 nM [13]. The structural diversity of bioactive diterpenoids in this species has led to new insights into opioid receptor function [2] as well as synthetic analogs whose potency, selectivity, and bioavailability exceed SalA [14–16]. However, most synthetic protocols depend on semi-synthesis from advanced precursors [17, 18] and are economically unfeasible from an industrial standpoint. Thus, a detailed understanding of the biosynthetic pathway responsible for the bioactive diterpenoids from *S. divinorum* is essential to explore their full clinical potential.

**Fig. 1:**
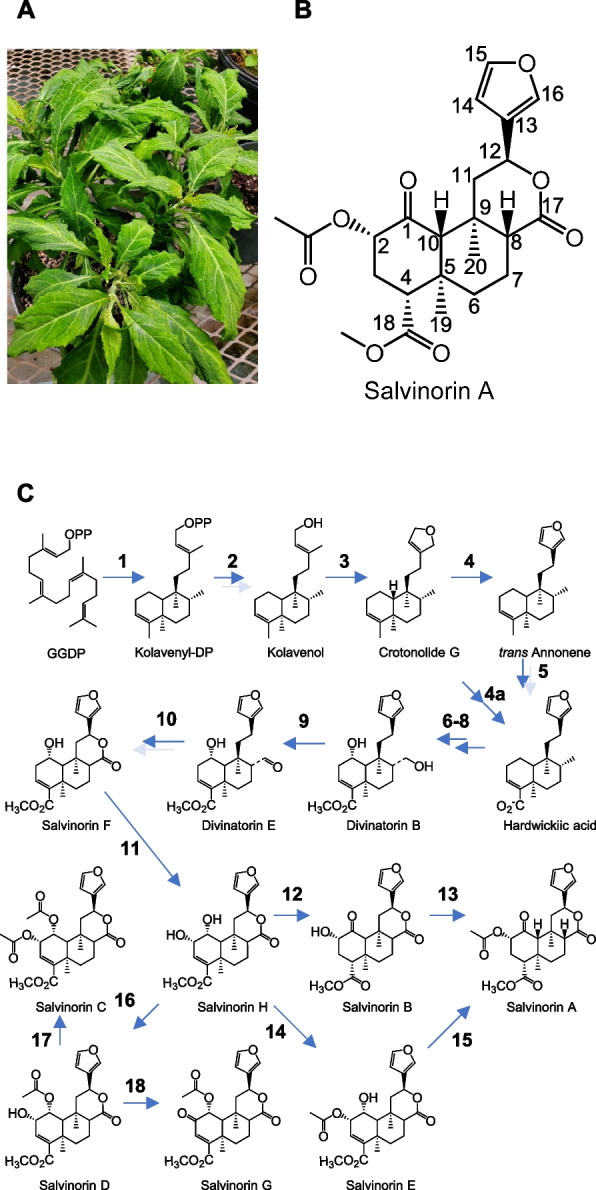
Diviner’s sage (*Salvia divinorum*) plants and salvinorin A proposed biosynthetic route. A, *S. divinorum;* B, Structure of salvinorin A showing numbering used in text. C, Proposed biosynthetic pathway based on biochemical characterization and intermediates isolated from *S. divinorum* leaves. The enzyme catalyzing reaction 1 has been described by Chen et al. 2017 and Pelot et al. 2017 while 3 was described in Kwon et al. 2022. Two dark arrows signify multi-enzyme sequences whose order is uncertain. Mixed light and dark arrows indicate sequences which may be catalyzed by single or multiple enzymes

The biosynthesis of typical labdane diterpenes, including the neoclerodane SalA, commences with an initial cyclization of GGDP by a class II diterpene synthase to a bicyclic product which retains the diphosphate group [4]. This is followed by a type I diterpene synthase, which removes the diphosphate group and may also introduce additional rings, double bonds, or hydroxyl groups [19]. In the case of SalA, GGDP is cyclized to kolavenyl diphosphate (KDP) by the type II diterpene synthase kolavenyl (or clerodienyl) diphosphate synthase (KDS) (Fig. 1; step 1) [20, 21]. KDP is subsequently dephosphorylated to kolavenol by an as-of-yet unidentified enzyme or enzymes (step 2). The dihydrofuran ring is next introduced by crotonolide G synthase (step 3) [22]. This cytochrome P450 introduces an O atom at C16 which initiates a nucleophilic attack to form the dihydrofuran ring, displacing the C15 alcohol as water. It is presently unclear whether the furan ring, which confers the KOR binding activity of SalA, is formed in the next biosynthetic step or later in the pathway. In case of the former, desaturation of the dihydrofuran ring of crotonolide G would produce *trans*-annonene (Fig. 1, step 4), but this intermediate has not been detected in *S. divinorum* to date. Alternatively, the furan ring may be completed after oxidation at C18 (step 4a). Evidence for the latter view consists of a partially characterized C1 (CYP728D25) and C18 hydroxylase (CYP728D26) from *S. divinorum* which act on crotonolide G as substrate [23]. This scenario may implicate salvidivins or salvinicins as intermediates in the SalA pathway, which naturally occur in *S. divinorum* [6]. However, they are not necessarily required for SalA biosynthesis and may constitute side products.

As originally proposed by Chen et al. (2017), the remainder of the SalA pathway may be postulated by structures of diterpenoids isolated from *S. divinorum* leaves [6, 13, 24, 25] (Fig. 1). Following (or perhaps preceding) assembly of the furan ring (steps 1–4), oxidation at C18 leads to hardwickiic acid (step 5). Hydroxylation at C1 and C17 and methylation at C18 yield divinatorin B (steps 6–8), although the exact order is uncertain. Subsequent dehydrogenation at C17 produces the aldehyde divinatorin E, and lactonization between C12 and C17, leading to the salvinorins, presumably stems from oxidations at C12 and C17 followed by elimination of water (steps 9–10). A hydroxylation at C2 of salvinorin F arrives at the vicinal alcohol salvinorin H, which may proceed to SalA by dehydrogenation at C1 (salvinorin B) and acetylation at C2 or by the same steps in reverse, passing instead through salvinorin E (steps 12–15). Acetylation at C1 instead of dehydrogenation to a ketone leads to salvinorins C, D, and G (steps 16–18).

*Salvia* (Lamiaceae:Nepetoideae: Mentheae: Salviinae) is the largest genus in the mint family with its over 1,000 species making up ~ 15% of the family. The intrageneric relationships within the *Salvia* genus have been refined using concatenated, coalescent, and network-based nuclear phylogenies [26], which support a common backbone topology of ~ 10 monophyletic subgenera. Assembled genome sequences for six economically important *Salvia* species have been reported. These include *danshen* (*S. miltiorrhiza*) [27, 28] and *nan danshen* (*S. bowleyana*) [29], sources of tanshinones, rosemary (*S. Rosmarinus*) [30], source of rosmarinic acid, chia (*S. hispanica*) [31], a rich source of several dietary supplements, and common sage (*S. officinalis*) [32], which is widely used in cooking. These genomes tend to be diploid and range in size from 400 to 600 Mbp except for the ornamental tetraploid *S. splendens* (807 Mbp) [33, 34].

The closest sequenced species which produces diterpenes similar to those found in *S. divinorum* is the traditional Chinese medicine herb skullcap (*Scutellaria barbata*) [35]. Skullcap accumulates clerodane diterpenes with anti-cancer applications [36], and its genome may offer clues to SalA biosynthesis. In the Lamiaceae, genes for diterpene biosynthesis tend to organize into discrete gene clusters consisting of microsyntenic blocks [37, 38] also referred to as biosynthetic gene clusters (BGC) [32]. A reference genome of *S. divinorum* would not only facilitate identification of candidate genes for the biosynthetic steps outlined above but also provide valuable insight into the diterpenoid biosynthetic gene complement of one of nature’s most medically important plant genera. Here we report a chromosome level reference genome for this species, an examination of the structure of its BGCs, and a detailed inventory of genes identified as candidates for involvement in the SalA pathway.

## Results

### Assembly of the *S. divinorum* genome

A preparation of genomic DNA purified from *S. divinorum* leaves showed an average length of > 50 kb according to DNA tape station analysis. A total of 62 Gb of consensus HiFi reads were generated on the PacBio Revio platform (N50 read length 14,336 bp; (Supplementary Fig. 1). *K*-mer distribution analysis of the reads predicted a genome size of ~ 546 Mb, low levels of heterozygosity (0.192%), ~ 53% repeat content, and a homozygous coverage of ~ 30x (Supplementary Fig. 2). Raw HiFi reads were assembled using hifiasm [39] and haplotypic duplication was removed from the haploid draft assembly with purge_dups [40]. Since many highly fragmented plastidic and mitochondrial DNA contigs were produced in the draft assembly, alternative approaches were taken to assemble the organellar genomes. Using ptGAUL [41], we assembled a complete, 150,899 bp circular chloroplast genome (Supplementary Fig. 3). Two circular mitochondrial genomes were assembled (272,775 bp and 44,943 bp) by providing hifiasm with subsampled mitochondrial DNA (Supplementary Fig. 4). A mitochondrial genome conformation of two circular chromosomes is a known feature of the *Salvia* genus [42, 43].

**Fig. 2:**
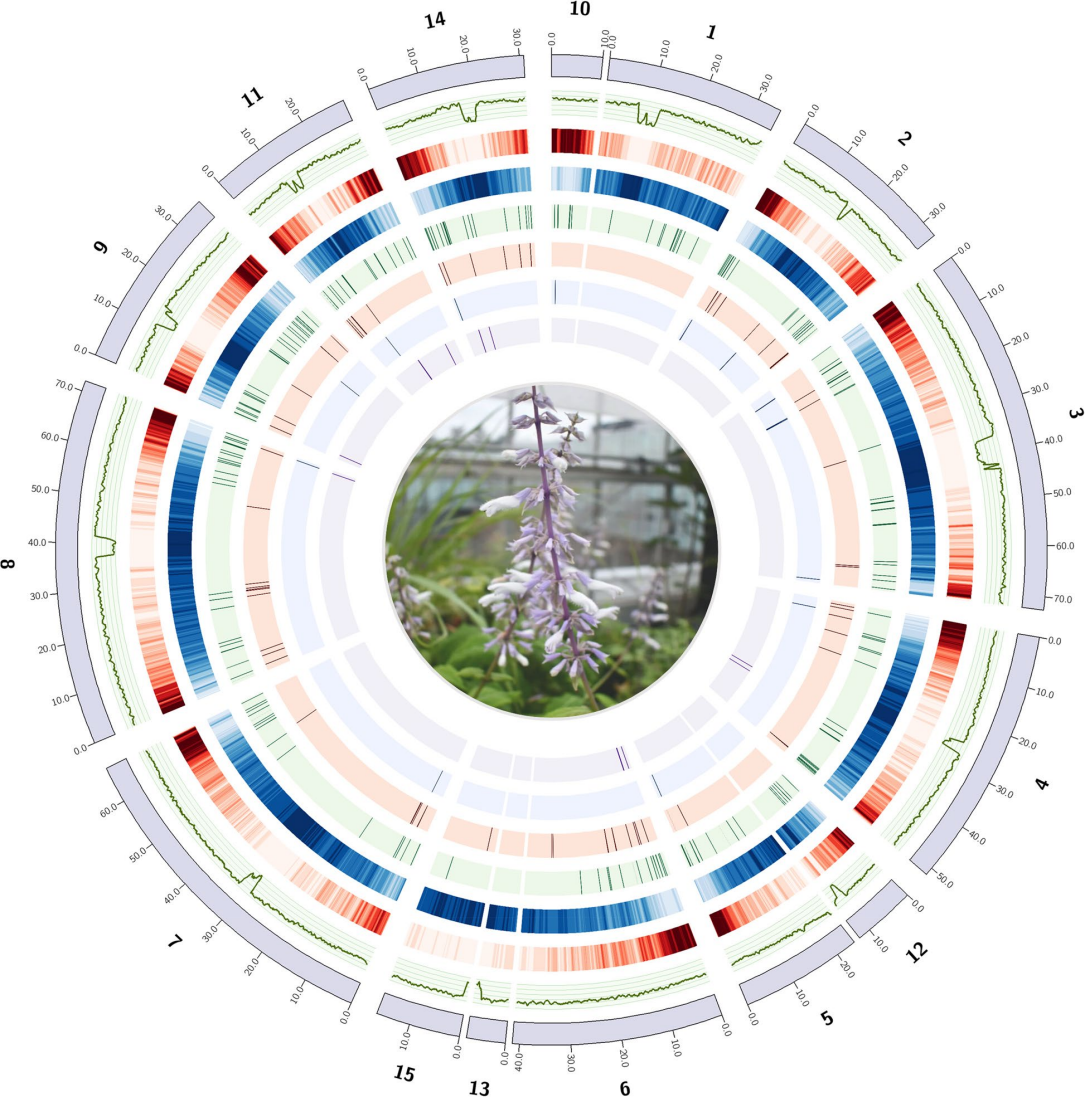
Circos plot of *Salvia divinorum* genome assembly. From outside to inside: scaffolds greater than 1 Mb (purple ideograms), GC% (green line, range displayed; 20–45%), gene density (500 Kb windows with 100 Kb step size), repeat content (500 Kb windows with 100 Kb step size), position of putative diterpene biosynthetic genes, including cytochrome P450s (light green), BAHD acyltransferase (light red), O-methyl transferases (light blue), diterpene synthases (light purple)

After filtering mitochondrial and plastidic contigs from the assembly, the resulting nuclear genome was 541 Mb (Fig. 2), consistent with *K*-mer predictions and genome sizes of closely related species. General assembly statistics are summarized in Table 1. The final assembly featured an N50 score of 42.4 Mb (L50 = 5) with a maximum contig length over 72 Mb and a median length of ~ 8.8 Mb. The BUSCO completeness of the genome was 98.4% when scored against the Embryophyta database [44]. Together, these statistics confirm that the assembly is of high quality with contiguity and completeness comparable to or exceeding the most recently sequenced *Salvia* genomes [29, 30, 32, 45].

**Table 1:** Genome assembly statistics for Diviner’s sage

Assembly statistics		Repeat content		Annotation statistics	
Total length	541,447,152 bp	Repeat content (%)	67.63	Gene number	34,514
Total contigs	28	Retroelements (%)	37.28	Mean gene length (bp)	2542.05
N50	41,401,808 bp	SINES (%)	0	Mean exon length (bp)	196.56
L50	5	LINES (%)	0.34	BUSCO^a^ (protein)	98.2%
Max contig length	72,546,039 bp	LTR (%)	36.94	[S:85.7, D:12.5]	
Median contig length	8,837,828 bp	Transposons (%)	3.17		
Contigs > 1 Mb (%)	50	Unclassified (%)	26.22		
Contigs > 100 Kb (%)	83.3				
BUSCO^a^ (genome)	98.4%				
[S:85.5, D:12..9]					

^a^Using embryophyta_odb10 database, *n* = 1614 genes

The repeat content in *S. divinorum* was 67.63%, with long terminal repeat (LTR) retrotransposons (RTs) accounting for 36.94% of the genome. Transposable elements constitute 3.17% while long interspersed nuclear elements (LINES) compose only 0.34%. As expected, regions of high repeat content were generally inversely associated with gene density, and the most repetitive regions of each chromosome were characterized by steep declines in GC content from ~ 39% to ~ 20% on average, likely indicative of centromeres (Fig. 2). It is also noteworthy that repeat content was much higher throughout the inner parts of each chromosome and gene density higher in the telomere proximal regions.

### Genome annotation

Based on ab initio prediction as well as evidence from mRNA sequencing and protein homology, the *S. divinorum* nuclear genome was predicted to have 34,514 genes, with an average gene length of 2,542 bp and exon length of 197 bp (Table 1). Among the predicted genes, 92.7% (31,995) were functionally annotated with protein products, Gene Ontology terms, and/or identifiers from external protein databases queried using InterProScan [46], eggNOG-Mapper [47] and funannotate [48]. The gene content of the *S. divonorum* genome was comparable to similarly sized diploid genomes of *S. miltiorrhiza* (530.97 Mb; 32,191 genes) [45] and *S. officinalis* (480 Mb; 31,713 genes) [32], but considerably lower than *S. bowleyana* (462.44 Mb; 44,044 genes) [29].

Within the 150,899 bp chloroplast genome of *S. divinorum*, we annotated 116 unique genes, 4 encoding rRNA, 30 encoding tRNA and 82 encoding proteins (Supplementary Fig. 3). The inverted repeat, small single copy and large single copy regions were 25,527 bp, 17,553 bp and 82,289 bp respectively. The structure of the *S. divinorum* chloroplast genome is consistent with the high conservation of plastid genome features within the genus [49]. In contrast, the structure of the mitochondrial genome is known to be diverse amongst *Salvia*, even at the intraspecies level [49]. In the two mitochondrial chromosomes of *S. divinorum*, we annotated 42 unique protein coding genes, 22 tRNA genes, and 3 rRNA genes (Supplementary Fig. 4). In spite of > 100-fold variation in mtDNA size across the plant kingdom, mitochondrial gene content varies little with about 60–70 genes found in mitochondrial genomes of most plant species [50].

### Chromosomal synteny with *S. splendens*

**Fig. 3:**
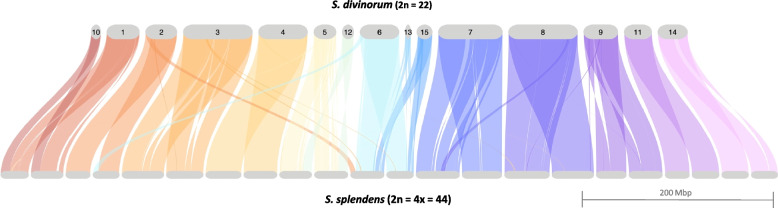
Riparian plot displaying syntenic relationship of Salvia divinorum and the tetraploid Salvia splendens [33]. Syntenic regions were identified using GENESPACE [84] which combines evidence from gene collinearity and sequence similarity

We analyzed orthologous gene order using GENESPACE [51] and observed a high degree of synteny between the genomes of the diploid *S. divinorum* [52] and the tetraploid *S. splendens* (Fig. 3)*,* suggesting few large-scale chromosomal rearrangements in spite of the whole genome duplication in *S. splendens*. The shared synteny between these genomes also permitted the identification of smaller contigs in our *S. divinorum* assembly that can likely be grouped with larger contigs in chromosomes, but have been broken either at repeats near the centromere (contigs 5/12, 15/13) or at other chromosomal regions (contigs 10/1; 6/13) (Fig. 2). Grouping these contigs together as chromosomes, our *S. divinorum* assembly has 11 nuclear chromosomes, as Reisfield reports (1993). Analyses of GC content, repeat density, and gene density indicate the presence of 11 centromeres (Fig. 2). Though these results strongly suggest linkages between the aforementioned contigs, we have elected to keep them separate in our assembly since we have no direct sequence evidence to scaffold contigs together.

### Genome evolution and phylogeny

**Fig. 4:**
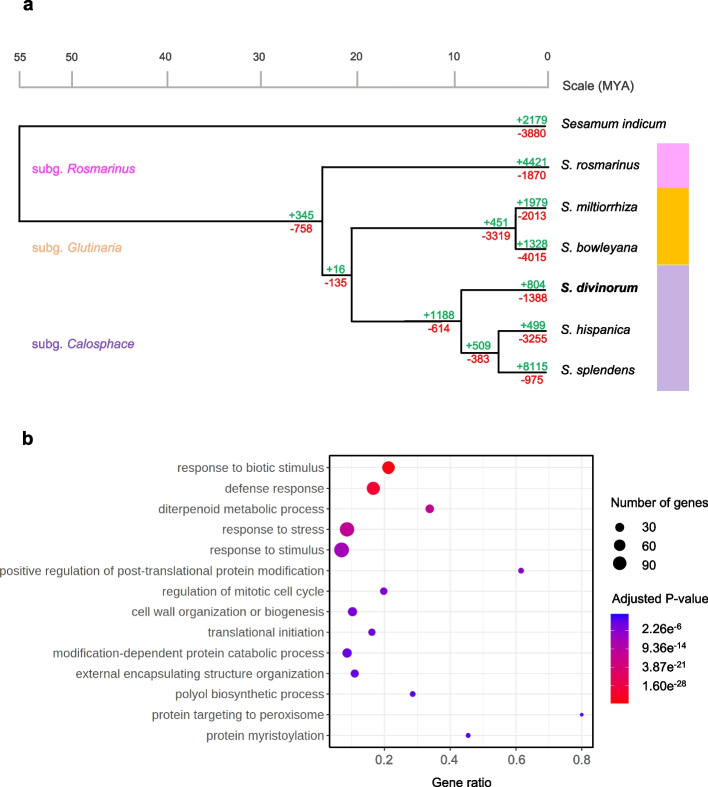
Genome evolution of *S. divinorum* and six related species and their divergence times. A, Maximum likelihood phylogeny constructed from a common set of protein orthologs (Emms and Kelly, 2019). Number of expanded and contracted gene families are shown in green and red, respectively, based on comparison of their orthogroups. The phylogeny was assembled using a JTT + CAT substitution model. *Sesamum indicus* served as outgroup. The colored boxes (right) indicate the Rosmarinus (pink), Glutinaria (orange), and Calosphace (green) subgenera. B, Significantly (*p* < 0.05) enriched GO Biological Process annotations in expanded gene families of *S. divinorum*

To infer the evolutionary history of *S. divinorum*, we constructed a maximum likelihood phylogeny (Fig. 4) of *S. divinorum* and six related species (*S. splendens*, *S. hispanica*, *S. rosmarinus*, *S. bowleyana*, *S. miltiorrhiza*, and *Sesamum indicum*) from protein orthologs identified with OrthoFinder [53]. The divergence of the monophyletic *Salvia* clade from the common ancestor of *Salvia* and the outgroup *Sesamum indicum* was estimated to occur ~ 55 MYA based on previous studies [30, 45]. Among *Salvia* species with available whole-genome sequences, *S. divinorum* was most closely related to *S. splendens* and *S. hispanica*, which are sister to each other, and diverged from their common ancestor with *S. divinorum* at an estimated ~ 8 MYA. The clade of *S. divinorum*, *S. hispanica* and *S. splendens* diverged from its common ancestor with the clade of *S. miltiorrhiza*, and *S. bowleyana* approximately 20 MYA. We estimate that the most distantly related *Salvia* species included in the analysis, *S. rosmarinus*, diverged from its common ancestor with other Salvia species ~ 23 MYA.

A total of 27,469 orthogroups were identified in the 7 plant species, and gene family evolutionary analysis was perfomed using CAFE 5. Within the *Salvia* genus, *S. splendens* showed the greatest expansion of these gene families, likely owing to its polyploidization, while *S. hispanica* showed the least (Fig. 4). Gene family expansion and contraction of *S. divinorum* was intermediate relative to other *Salvia* species, with 804 expansions and 1388 contractions. GO enrichment analysis demonstrated that significantly (*p* < 0.05) expanded orthogroups in *S. divinorum* were enriched in genes involved in biological processes such defense against other organisms, stress response, and diterpene metabolism (Fig. 4B). All 26 genes within an expanded family, annotated by the GO term ‘diterpene metabolic process’, are class I terpene synthases (*TPS*). Twenty four belong to the *TPS*-a subclade with similarity to sesquiterpene synthase-like enzymes while the other 2 are kaurene synthases in the *TPS*-e/f subclade (Supplementary Data Set 1A). Considering reports of sesquiterpene synthases acquiring plastid signal peptides and becoming active in diterpene pathways [54, 55], we used TargetP 2.0 [56] to predict the presence of N-terminal presequences in all TPS proteins. However, none of the sesquiterpene synthase-like enzymes in expanded gene families were predicted to be plastid localized (Supplementary Data Set 1B). Enriched molecular functions within expanded orthogroups included binding to ADP, ions and heterocyclic compounds, as well as TPS and O-methyltransferase activity (Supplementary Fig. 5).

### Diterpene biosynthetic genes in the *S. divinorum* genome

**Fig. 5:**
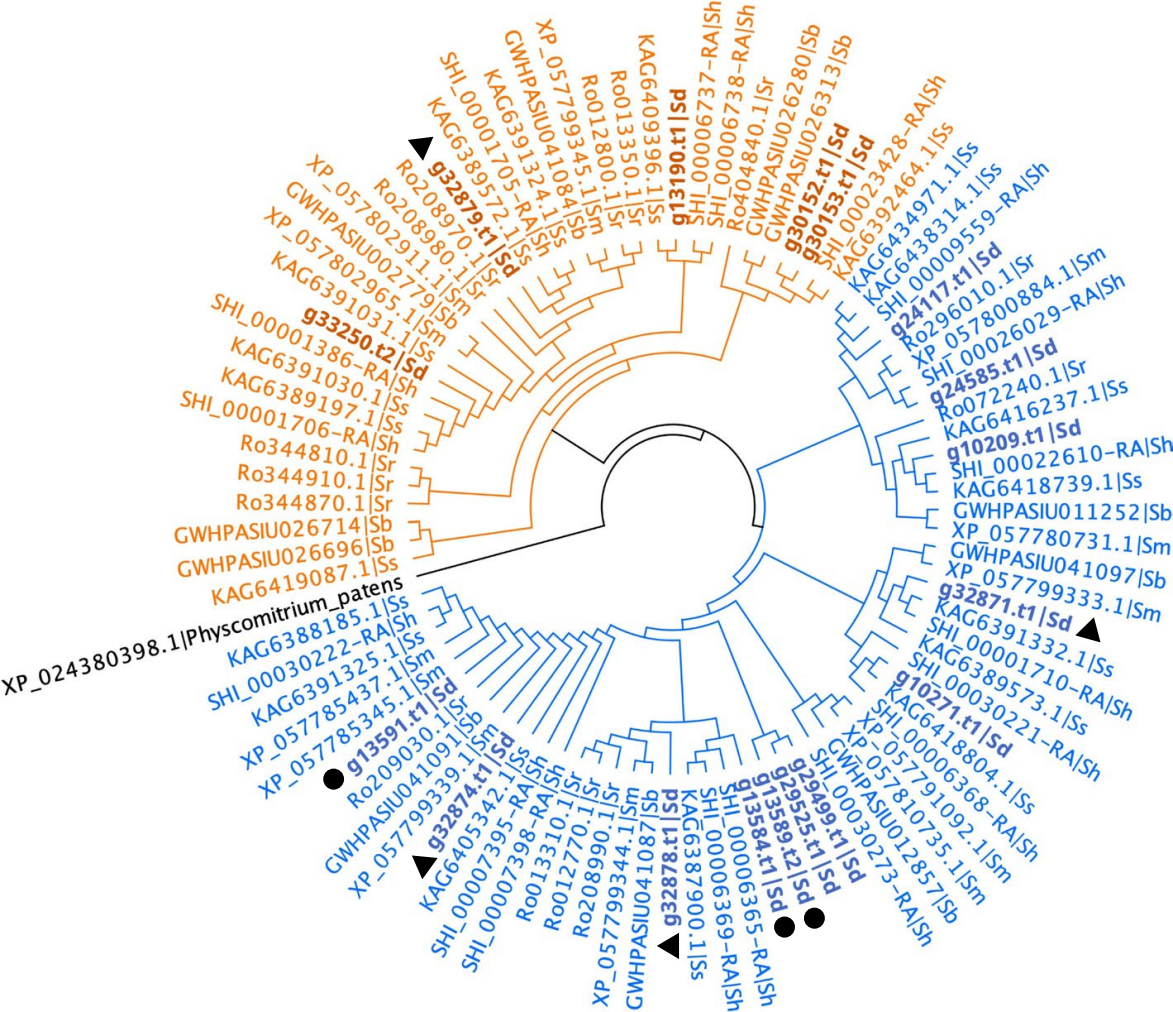
Neighbor-joining phylogeny of class II (blue) and class I (orange) diterpene synthases in *S. divinorum* (Sd; bold), *S. rosmarinus* (Sr), *S. miltiorrhiza* (Sm), *S. splendens* (Ss) and *S. bowleyana* (Sb). Rooted on the outgroup, ancestral bifunctional class I/ II diTPS *Physcomitrium patens* ent-kaurene/kaurenol synthase. Circles and triangles indicate presence in the contig 6 and contig 14 biosynthetic gene clusters, respectively as shown in Fig. 6

We observed a total of 107 TPS genes in the genome that consisted of 84 *TPS*-a/b/g, 12 *TPS*-c (type II), and 11 *TPS*-e/f [57–60] (Supplementary Data Set 1B). To gain insight about the subcellular localization of TPS proteins, TargetP 2.0 was used to predict the presence of N-terminal transit peptides (Supplementary Data Set 1B). A total of 17 putative diterpene synthases were detected which included 12 class II and five class I (Supplementary Data Set 1C). We constructed a neighbor-joining tree of diterpene synthases in the genomes of five *Salvia* species, using the ancestral bifunctional class I/ II diTPS ent-kaurene/kaurenol synthase of *Physcomitrium patens* as an outgroup (Accession: XP_024380398.1) (Fig. 5).

Cytochrome P450s (CYPs), which introduce oxygen functional groups into the core structure, are expected to play multiple roles in the SalA pathway. A total of 392 CYP genes were detected (Supplementary Data Set 2) that encode 422 predicted transcripts including isoforms (Supplementary Data Set 1D). Nine CYP families were represented (Supplementary Fig. 6). The majority of CYPs were in the CYP71 clan, which is known for its involvement in diterpenoid biosynthesis [5].

Our examination of the genome for additional enzymes likely to be involved in the SalA pathway predicted 69 putative alcohol dehydrogenases (ADHs) (Supplementary Data Set 1E), 39 SAM-dependent O-methyl transferases (OMTs) (Supplementary Data Set 1F), and 105 BAHD acyl transferases (BATs) (Supplementary Data Set 1G). These classes of enzymes were found on each chromosome in the *S. divinorum* genome, showing no strong positional bias beyond the large-scale tendency for genes to be at the ends of chromosomes (Fig. 2).

**Fig. 6:**
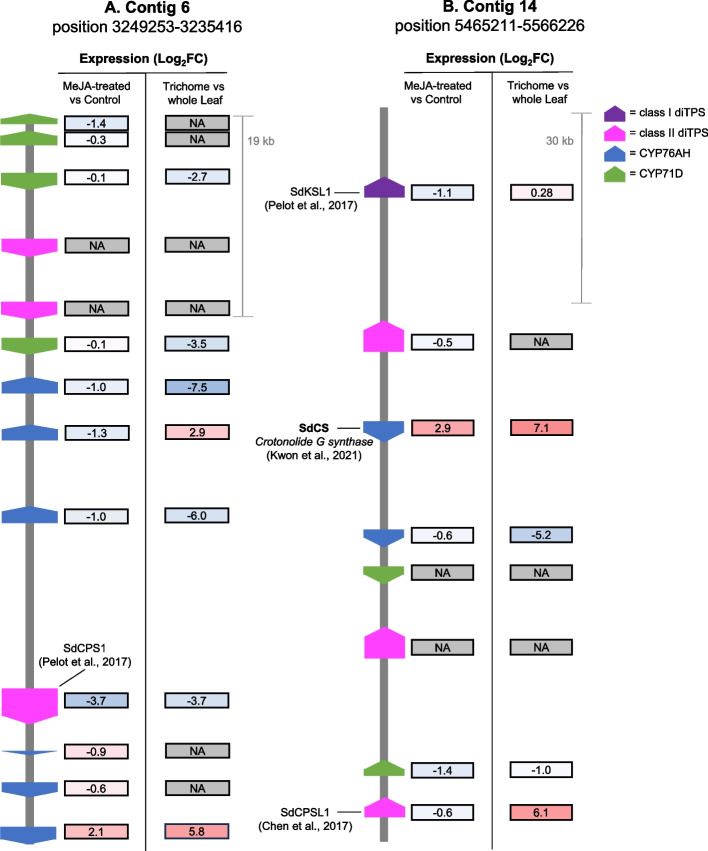
Diterpene biosynthetic gene cluster on contigs 6 (a) and 14 (b) in the *Salvia divinorum* genome. Genetic map shows position and class of diterpene biosynthetic genes. MeJA-treated vs control represents the log_2_fold change (FC) in expression of methyl jasmonate-treated plants relative to control plants, while trichome vs whole leaf shows log_2_FC in expression in trichomes [20] relative to whole leaves. Colors are scaled independently for each RNA-sequencing data set and depict the magnitude of expression change relative to all genes in enzyme classes postulated to participate in SalA biosynthesis (Supplementary Data Set 1 J)

There is evidence in the mint family that genes in the same secondary metabolic pathway cluster together in the genome [37]. Two BGCs identified on contigs 14 and 6 were enriched in diterpene synthases and CYP450s (Fig. 6; Supplementary Data Sets 1H and 1I). Among the CYPs in the BGC on contig 14 was crotonolide G synthase [22], an enzyme highly expressed in glandular trichomes which catalyzes the formation of crotonolide G (dihydrofuran neoclerodane) from (-)-kolavenol. Other CYPs in the cluster included one CYP76B (g32876), one CYP71BE (g32875) and one CYP71D (g32873).

Putative diterpene synthases identified in BGCs included six class I and one class II enzyme. Despite clustering with crotonolide G synthase on contig 14, the class I diTPS kaurene synthase-like 1 (SdKSL1) was reportedly unable to convert CLDP into (-)-kolvaenol for SalA biosynthesis [21]. The class II diTPS, copalyl-diphosphate synthase-like 1 (SdCPSL1), located in the same BGC, was previously predicted not to produce (-)-kolavenol diphosphate [(-)-KPP] in the SalA pathway based on its similarity to class II diTPSs that synthesize ( +)-copalyl diphosphate [21]. However, copalyl-diphosphate synthase 1 (SdCPS1), found in the BGC on contig 6, grouped phylogenetically with ( +)-KPP synthases (Fig. 6; g13584) but was shown to have (-)-KPP synthase activity [20]. Though this finding provides incentive for functional investigation of SdCPSL1, SdCPS1 is predominantly expressed in roots and is not thought to be involved in SalA biosynthesis [20]. Other diterpene synthases and CYPs within the biosynthetic gene clusters on contigs 6 and 14 have not yet been characterized, but do not appear to be highly enriched in trichomes (Supplementary Data Set 1 J). Notably, copalyl-diphosphate synthase 2 (SdCPS2; g10209), which catalyzes the first step in the proposed SalA biosynthetic pathway [20, 21], is isolated on chromosome 4, and no coding sequence for biosynthetic enzymes was found within 200 Kb of this gene.

### Glandular trichome-specific and methyl jasmonate-responsive transcript expression

The assembled *S. divinorum* genome allowed us to profile the enrichment of RNA transcripts putatively involved in SalA biosynthesis in enriched tissue (glandular trichomes) or in response to induction from the defense hormone methyl jasmonate (MeJA). RNA-seq analysis from all tissue sources corroborated 93% of the predicted proteins. In response to MeJA induction, 7% of annotated TPS genes were significantly upregulated (*p* < 0.05); however, none of them was predicted to be a diterpene synthase (Supplementary Data Set 1B, 1C, and 1 J). Among CYP450 genes, 7% were also enriched following MeJA induction. Significant upregulation was also observed for 9, 14, and 15% of ADH, BAT and SAM-dependent OMT genes, respectively (Supplementary Data Set 1E, 1F, 1G, and 1 J). Confirming the effectiveness of MeJA treatment, we observed the upregulation of fatty acid hydroperoxide lyase (g11225), allene oxide cyclase (g206), and the JASMONATE ZIM-domain (JAZ) transcriptional repressor, JAZ1 (g116446), which aligns with the common response in MeJA-treated plants. Consistently, the BTB/POZ domain-containing, nonexpressor of pathogenesis-related genes 1 (NPR1; g30536), a key activator of salicylic acid (SA)-mediated immune responses was significantly downregulated in MeJA treated plants.[61]

Expression of genes in diterpene metabolic gene clusters was not clearly affected by MeJA treatment. Among genes in the two identified clusters (Fig. 6), only crotonolide G synthase on chromosome 14 (which synthesizes crotonolide G in the SalA pathway) was significantly upregulated (Log_2_fold change (FC) = 3.0; *p* < 0.05). However, three CYP450 genes in the cluster on contig 6 were significantly downregulated (Log_2_FC = -3.7, -1.3, -1.0; *p* < 0.05). Despite induction of crotonolide G synthase and another CYP450 implicated in the SalA pathway (CYP728D26 or g5204) [23] with Log_2_FC = 2.2 (p ~ 0.01), expression of SdCPS2, the class II diTPS catalyzing the first committed step in the SalA pathway, was not significantly altered by MeJA (Log_2_FC = 0.6). Among the four genes with an established or putative role in the SalA pathway, only crotonolide G synthase demonstrates statistically significant induction by MeJA (Supplementary Table 1), indicating that not all enzymes in the SalA pathway are uniformly co-expressed following MeJA treatment.

Similarly, clustered diterpene biosynthetic genes were not uniformly enriched in the trichomes (Supplementary Data Set 1 J). The majority of genes in these clusters were downregulated in trichomes. RNA-seq analysis showed that aside from aside from crotonolide G synthase, no CYP in this cluster was upregulated in trichomes (Supplementary Data Set 1 J). Two CYP76Bs of unknown function in the contig 6 BGC were enriched in trichomes (g13581 and g13586; Log_2_ FC > 2). However, genes implicated in the SalA pathway, regardless of position in the genome, were uniformly highly expressed in trichomes (Supplementary Table 1). These results corroborate the observations above that SalA pathway genes are neither uniformly induced by MeJA treatment nor clustered together in the genome.

## Discussion

### Most SalA genes are not associated with BGCs

*S. divinorum* is a medicinal plant with a long history in shamanistic healing ceremonies of the Mazatec people of Oaxaca, Mexico. Its use in such rituals is due to the psychoactive diterpene SalA, which accumulates in glandular trichomes of *S. divinorum* aerial tissues. Despite its potential medical applications in the treatment of chronic pain, addiction, and depression, only two steps in the SalA biosynthetic pathway are currently known. Here we have generated a high-quality reference genome sequence which will serve as a guide to facilitate the complete elucidation of the SalA pathway and better understand the unique secondary metabolism of the *Salvia* genus.

Our genome assembly is highly contiguous with N50 values that mark a substantial improvement upon other recently published *Salvia* genomes [30, 33]. The high BUSCO completeness scores of this assembled genome (98.4% and 98.2% at the genome and protein levels, respectively) also signify that the assembly is of high quality with significant value for the goal of elucidating the SalA pathway. This assembled genome sequence is among the highest contiguity of any *Salvia* genome published to date.

The repeat content of *S. divinorum* was noticeably higher than that of *S. splendens* (47.99%) [33], *S. miltiorrhiza* (between 54.44% [28] and 56.65% [45]), or *S. bowleyana* (58.7%) [29] but similar to *S. officinalis* (61.67%) [32]. Another report placed the repeat content of *S. miltiorrhiza* even closer to *S. divinorum* (64.84%) [27]. Within the genus, the genome of *S. rosmarinus* currently possesses the highest repeat content at 72.72% [30].

Genes for diterpene metabolism have been known to form clusters in the Lamiaceae [37], and a recent comparison of several *Salvia* genomes confirmed that BGCs are a typical feature of genomes in this family [30]. In the *S. divinorum* genome, we identified two BGCs located on contig 6 and contig 14, each enriched in CYPs and diterpene synthases. Notably, the BGC on contig 14 contains crotonolide G synthase, the CYP which forms the dihydrofuran ring on C16 of the neoclerodane backbone in salvinorin biosynthesis [22]. In addition to crotonolide G synthase, sequences for three other CYPs were identified in this BGC, but only two were expressed. CYPs within the cluster were differentially regulated, as only crotonolide G synthase showed a significant increase in expression after MeJA treatment, while the other two expressed CYPs were slightly downregulated. Similarly, two of the three diTPSs in this cluster were expressed, and both showed slight downregulation in response to MeJA. The other identified BGC, on contig 6, contained 10 CYPs, 3 of which were significantly downregulated by MeJA, while one was strongly upregulated in response to MeJA (g13581; Log_2_FC = 2.12; *p* = 0.07). This transcript was also significantly enriched in trichomes (Log_2_FC = 6.14) and therefore represents a promising candidate for involvement in the SalA pathway. Only one diTPS in this cluster (SdCPS1; g13584) was expressed, and it was significantly downregulated both in trichomes (Log_2_FC = -3.74) and after MeJA treatment (Log_2_FC = -3.71).

Genes in BGCs are often co-regulated. For instance, the BGCs involved in paclitaxel synthesis in *Taxus chinensis* showed tightly coordinated expression patterns in tissues and in response to jasmonate [62]. Differential regulation of genes within diterpene gene clusters has also been observed in *S. officinalis* [32]. In the latter, a single BGC contained two pairs of class I and class II diTPS, and each was independently co-expressed in roots and shoots despite membership in separate diterpene biosynthetic pathways in these organs [32]. We speculate that in *S. divinorum*, the pair of class I (SdKSL1) and class II diTPSs (g32873) in the BGC of contig 14 may be part of a diterpenoid pathway in roots, as SdKSL1 was shown to be expressed in roots [20] and the pair showed coordinated downregulation in response to MeJA (Supplementary Data Set 1 J). This would suggest that crotonolide G synthase may have dual roles in biosynthesis of SalA and root diterpenoids, as has been previously documented for CYP76AHs in the BGC of *S. officinalis* [32]. SdCPS2 in contrast, which catalyzes the committing step in the SalA pathway, is isolated on chromosome 4. These observations lead us to conclude that genes encoding enzymes in the SalA pathway are not exclusively found in diterpene metabolic gene clusters. Similarly, paclitaxel biosynthesis involves two discrete BGCs as well as many genes not found in BGCs [62]. But unlike the known genes for SalA biosynthesis, most are found in a small region of a single chromosome. Thus, the organization of clerodane biosynthetic genes in *S. divinorum* more closely resembles that of skullcap, wherein many clerodane biosynthetic genes are not associated with BGCs but appear instead to be the products of gene duplications of genes that are [35]. This is consistent with the assertion that BGCs may serve as toolboxes for recruitment of new catalysts in emergent secondary metabolic pathways.

### Insights into kolavenol formation

Class II diTPS enzymes catalyze the rearrangement of geranylgeranyl diphosphate (GGDP) with retention of the diphosphate group. They are all derived from an ancestral diTPS sequence which produces *ent-*copalyl diphosphate, a precursor to gibberellin [59]. Examples which produce copalyl diphosphate, clerodienyl diphosphate, and isokolavenyl diphosphate have been described [19, 63, 64]. However, no class I diTPS participating in a clerodane diterpenoid biosynthetic pathway has been identified to date [35]. The conversion of KDP to kolavenol (Fig. 1; step 2) therefore remains unclear. Several possibilities have been proposed. Extensive transcript mining has not thus far not produced a type I diTPS that carries out the conversion of KPP to kolavenol. Three type I diTPS (SdKSl1-3) were previously identified [21]. We found evidence for two additional type I diTPS sequences in the *S. divinorum* genome. One does not appear to be expressed (g33250). The other, g30152, is 97% identical to SdKSl and shows low expression relative to other diTPSs. However, it does appear to be predominantly expressed in trichomes, warranting future functional characterization.

An alternative explanation for conversion of KDP to kolavenol is the Nudix hydrolase family of enzymes, which have found an unexpected place in terpene metabolism over the past several years [65–69]. Nudix hydrolases act on diphosphate substrates through a phosphohydrolase mechanism, removing a single phosphate group [70]. Although mostly involved in housekeeping metabolism, one subclade of Nudix hydrolases preferentially acts on terpenoid substrates [71]. It has been suggested that conversion of prenyl diphosphate substrates to their monophosphate analogs increases their susceptibility to non-specific phosphatases to yield the corresponding alcohol [65]. We searched the *S. divinorum* genome for Nudix hydrolases which might convert KPP to KP and identified three genes which clustered within the terpene modifying subclade of plant Nudix hydrolases (g224, g225 and g7372) (Supplementary Fig. 7 and Supplementary Data Set 1 K). None of these sequences occurred within the two BGCs identified here (Fig. 6). Two Nudix sequences, g224 and g7372, were highly upregulated following MeJA treatment (Log_2_FC = 2.07 and 2.16, respectively), while no other Nudix hydrolase showed a Log_2_FC greater than 1.17 (Supplementary Data Set 1 J). Additionally, g225 was highly expressed in glandular trichomes relative to whole leaves (Log_2_FC = 6.67), and predicted to have a chloroplast transit peptide. Together, these results provide a strong rationale for further investigating a possible role for Nudix hydrolases in the SalA pathway.

### Clinical and evolutionary implications of the sala pathway

The use of psychoactive substances to treat resistant conditions such as post-traumatic stress disorder (PTSD), depression, and addiction has received increasing mainstream medical attention in recent years. Multiple clinical trials have reported positive results using psilocybin, ketamine [72], and ayahuasca to treat chronic pain and mental disorders [73, 74]. As decriminalization continues to improve access for clinical evaluation of psychoactive substances, their potential to alleviate major depression and other neurological conditions enjoys increasingly broad support [75]. Medicinal plants have long been the source of psychoactive and analgesic substances, and new drugs procured from Indigenous medicine traditions have the benefit of centuries of informal clinical testing that attest to their efficacy and safety. *S. divinorum* represents a particularly understudied example of this. Roth et al. [76] recently demonstrated that SalA, despite its exquisite affinity for the KOR, has no appreciable affinity for serotonin or other neuroreceptors. SalA as a research tool highlights the prominent role of the KOR as an important modulator of perception and emphasizes its clinical potential for both understanding and treating mental disorders [76]. SalA’s highly selective agonism of KOR (K_i_ of 4 nM) [77] offers a critical alternative to traditional pain relief methods that rely on μ opioid receptor (MOR) agonism, which leads to dependency and addiction. Unlike MOR agonism, KOR activation by SalA is not associated with addictive behavior and may, in fact, have anti-addictive properties [7, 12]. The availability of genomic resources and structural genes presented here will improve access to SalA and its derivatives for clinical evaluation of these properties.

The *Salvia* genus is a rich source of medicinal plant species and compounds. One of the major questions arising from studies of medicinal terpenoids is how and why these pathways arose through evolution. Bergman et al. argue that the fortuitous uses plant terpenoids find in medicine is the result of two forms of evolutionary pressure [78]. Firstly, the common folds between plant and animal proteins made from a common set of amino acids suggest that a natural product made by plant biosynthetic enzymes may have a chemical structure that interacts favorably with human proteins compared to synthetic analogs (biochemical pre-selection). Secondly, plants have faced significant selective pressure over time to protect themselves against insect herbivores via defense compounds [79], and exploiting insect neuroreceptors has been among the most successful strategies. Humans neuroreceptors share a high degree of similarity to their insect homologs due to our evolutionary link to all animals. This evolutionary pre-selection for plant natural products with biological activity towards animal neuroreceptors may in part explain why they produce successful lead compounds at a higher frequency than constituents of combinatorial chemical libraries [80, 81].

## Conclusions

We have presented the first assembly and annotation of the *S. divinorum* genome. This high quality genome will serve as a valuable resource for future studies aiming to elucidate steps in the biosynthetic pathway of the potent *K*-opioid receptor agonist, SalA. The sequenced genome will provide a framework for leveraging the medicinal potential of *S. divinorum.*

## Methods

### Genomic DNA isolation, sequencing, and assembly

High molecular weight DNA was extracted from frozen, dark-treated *S. divinorum *leaf tissue using a standard cetyltrimethylammonium bromide (CTAB) / phenyl:chloroform:isoamyl alcohol method. To confirm identity of the species, gas chromatography – mass spectrometry (GCMS) analysis of the same leaf material was performed. For GCMS analysis, 10 mg dried *S. divinorum* tissue was extracted once with 0.5 mL acetonitrile, filtered, and injected onto an Agilent 7890C gas chromatograph fitted with a VF-5 ms capillary column (30 m × 250 μm i.d. × 0.25 μm film thickness) and coupled to an Agilent 5977C mass selective detector. The injection was performed with a split ratio of 1:25 and isothermal conditions at 100 °C for 1 min, followed by a temperature gradient at 12 °C min^−1^ to 300 °C and a final hold time of 12 min. The analyzer scanned from *m/*z 50–550 with an electron impact energy of + 70 eV. The leaf extract produced a peak with the same retention time as an authentic salvinorin A standard (Millipore-Sigma), confirming its identity as *S. divinorum* (Supplementary Fig. 8).

For DNA extraction, ~ 0.5 g of ground leaf tissue was suspended in 2 mL of 65° C CTAB buffer with 5 μL each of proteinase K (20 mg∙mL^−1^ stock) and RNAse A (100 mg∙mL^−1^ stock) and incubated at 65° C for 30 min. After cooling to room temperature, 2 mL phenol:chloroform:isoamyl alcohol (25:24:1) were added to the solution and mixed by inversion for 5 min at 4° C, then centrifuged at 1500 × g for 5 min. The aqueous phase was isolated, and re-extracted as above, this time with an equal volume of chloroform:isoamyl alcohol (49:1). The aqueous phase was collected, and DNA was precipitated by the addition of 2 × volume of ice cold 100% ethanol. DNA was pelleted by centrifugation at 20,000 × g for 1 min, and washed with 70% ethanol, before a final resuspension in ultrapure H_2_O. A 1 × volume of SPRIselect bead-based reagent (Beckman Coulter, Inc.) was used for DNA cleanup and purification prior to library preparation. SMRTbell library preparation was performed by The Centre for Applied Genomics at SickKids Hospital (Toronto, Canada) and Pacbio Hifi sequencing was conducted on the Revio SMRTcell platform.

To estimate genome size, Jellyfish v2.2.10 [82] was used to count canonical (-C) 21 bp k-mers from the PacBio HiFi reads, and the resulting k-mer frequency histogram was provided to GenomeScope [83] to fit a model for the prediction of the genome length, heterozygosity, and repetitiveness.

Raw Pacbio HiFi reads were assembled using two validated softwares: (1) hifiasm v0.16.1 [39] and (2) Flye v2.8.1 [84] with default parameters. The haploid assembly generated by hifiasm (458 contigs; N50 = 41 Mb) was more contiguous than the Flye assembly (1760 contigs; N50 = 11.1 Mb), and was therefore selected for further processing and subsequent analyses.

To remove erroneous allelic duplication and low coverage contigs from the assembly purge_dups v1.2.5 [40] was used after self-alignment using Minimap2 v2.22 [85]. A single circular chloroplast chromosome was assembled from HiFi reads using ptGAUL [41]. ptGAUL uses a reference chloroplast genome from a related species to select HiFi reads originating from plastid DNA, downsamples the reads to ~ 50 × coverage, and assembles them with Flye. We used a reference chloroplast genome of *S. madrensis,* a close relative of *S. divinorum*, with an estimated chloroplast genome size of 150 Kb. A similar approach was used to assemble the mitochondrial genome; we first mapped HiFi reads to a concatenated FASTA file of *S. splendens* and *S. miltiorrhiza* mitochondrial genomes using Minimap2, and filtered mappings for length > 1,000 bp and number of residue matches/length > 0.7. After downsampling the mapped reads to ~ 50 × coverage (using an estimated mitochondrial genome size of 300 Kb), we assembled them with hifiasm, which produced two non-redundant circular mitochondrial DNA chromosomes. To filter redundant organellar DNA contigs from the final assembly, we mapped assembled contigs to the organellular genomes and removed those which had a mapping length of > 99% contig length. To assess completeness, the assembly was queried against the embryophyta_odb10 database using BUSCO v5.6.1 [86] in genome mode. All code used in genome assembly, annotation, and functional identification of BGCs are available in Supplementary Methods file 1.

### Structural annotation

For improved gene-model prediction, we first softmasked the assembled genome. A species-specific repeat library was generated using RepeatModeler v2.0.5 [87] and merged with eudicot repeat database from RepBase v. 20,240,126. To softmask repeats, the joined library was provided as input to RepeatMasker v4.1.6 [88].

To predict gene models, we combined evidence from species-specific RNA-seq and proteins from related plant species. First, we ran BRAKER v3.0.8 [89] in RNA-only mode using raw *S. divinorum* RNA-seq reads obtained from whole leaves and isolated trichomes [20] (Supplementary Data Set 1L), as well as the control and MeJA-induced leaves generated in this study. Next, we created a protein database consisting of all Viridiplantae proteins from OrthoDB v11 [90] merged with Lamiaceae proteins downloaded from UniProt (20,240,220). The resulting 5,401,793 plant proteins were used as input to BRAKER, running in protein-only mode. We then combined evidence from protein-only and RNA-only BRAKER modes using TSEBRA [89] and applied the script agat_convert_gxf2gxf.pl from AGAT v1.0.0–pl5321hdfd78af_0 [91] (https://zenodo.org/records/5834795) to create a GFF3 file from the TSEBRA-generated GTF. To evaluate gene prediction, we queried the predicted protein sequences against embryophyta_odb10 using BUSCO v5.6.1 in protein mode.

We used GeSeq to annotate the organellular genomes [92] and produced graphical maps using OGDRAW v1.3.1 [93]. We used BLAT search for prediction of coding sequence, rRNA and tRNA, with additional tRNA annotation by tRNAscan-SE v2.0.7. We provided GeSeq with the reference mitochondrial genome of *Arabidopsis thaliana*, and reference plastid genomes of *S. splendens*, *S. miltiorrhiza*, and *S. hispanica* (Supplementary Data Set 1L). For plastome annotation, we used profile searches of Embryophyta chloroplasts by HMMER and Chloe v0.1.0 to supplement BLAST and predictions of coding sequences and rRNA.

### Functional annotation and biosynthetic gene cluster analysis

Functional annotation of genes was performed using eggNOG-Mapper v2 [47] and InterProScan v5.66–98.0 [46] which queried 14 protein databases and assigned Gene Ontology (GO) terms. EggNOG-Mapper and InterProScan outputs were used as input to funannotate v1.8.14 [48], which combined the annotations and queried additional databases CAZy, MEROPS and BUSCO. As described by Santangelo et al., [94] (https://github.com/James-S-Santangelo/dcg/tree/main), if a protein was annotated as ‘hypothetical protein’, but was assigned a fully resolved enzyme commission (EC) number, the annotation was replaced with the EC number’s product in the ExPASSY Enzyme database [95].

Based on the expected enzyme classes participating in the SalA pathway (Fig. 1), candidate TPSs, alcohol dehydrogenases, CYP450s, BAHD acyltransferases, SAM-dependent O-methyltransferases and Nudix hydrolases in *S. divinorum* were identified using representative Pfam and InterPro domains (Supplementary Data Set 1L). CYPs were putatively classified into clans using a protein–protein BLAST v2.5.0 [96] search of predicted *S. divinorum* CYPs against a database of 9739 plant CYPs previously classified by Dr. David Nelson (https://drnelson.uthsc.edu/plants) (Supplementary Data Set 2). To identify putative diterpene synthases, we performed a BLAST search of a class II diTPS, SdCPS2, (Accession: A0A1S5RW73.1), a class I diTPS, SdKSL1 (Accession: XP_057799345.1), and a bergamotene (sesquiterpene) synthase (Accession: XM_048123031.1) against a database of all predicted *S. divinorum* proteins. To obtain the list of diTPSs, we filtered genes in which the sesquiterpene synthase was the top hit, and applied a bit score cutoff of 45, which corresponded to a maximum e-value of 1.4e-21. Neighbor-joining trees were assembled using Geneious Prime 2021.2.2 (https://www.geneious.com). Presence of transit peptides was predicted using TargetP-2.0 [56].

To complement the class-specific search above, we further refined our list of candidate genes in the SalA pathway by searching for BGCs. We supplied PlantiSMASH [97] with our *S. divinorum* genome assembly fasta file and GFF file using default settings to identify putative BGCs that might contain genes for secondary metabolite biosynthesis.

### Genome evolution and gene family expansion and contraction

Published protein sequences of five *Salvia spp.* and *Sesasmum indicum* (Supplementary Data Set 1L) were obtained and used to make evolutionary inferences. After filtering protein sequences to include only the longest isoforms, orthogroups of *S. divinorum* and the six additional plants were identified using OrthoFinder v2.5.5 [53] with the following parameters: -M msa -S diamond -A mafft -T fasttree. A maximum likelihood species tree rooted on *Sesamum indicum* was inferred by FastTree [98] using a JTT + CAT substitution model. The tree was made ultrametric using the OrthoFinder script make_ultrametric.py with a root divergence time (-r) of 55 MYA, as determined previously [30]. 

Based on the obtained tree file and orthogroup gene counts, CAFE 5 [99] was used for determination of gene family expansions and contractions. Prior to analysis, the python script clade_and_size_filter.py, included with CAFE 5, was used to remove 22 of the 22,234 total orthogroups in which a single species had > 100 gene copies. Significance of gene family expansions and contractions was determined using a threshold of *P* < 0.05. Gene ontology (GO) enrichment analysis was performed for genes within significantly expanded gene families in *S. divinorum* using GOATOOLS [100] with GO annotations obtained from InterProScan [46]. 

We assessed pairwise synteny between *S. divinorum* and *S. splendens* using GENESPACE v1.3.1 [51] which employs MCScanX [101] to detect regions with conserved gene order and OrthoFinder [53] to identify paralogs and orthologs within these syntenic blocks.

### RNA-seq analysis

RNA transcript enrichment was calculated for glandular trichomes and, on the whole leaf level, in response to induction by MeJA. In the first experiment, RNA from isolated trichomes was compared to that of leaves. Preparation of trichome-enriched RNA-Seq analysis was performed as previously described [20] and deposited in NCBI (Accession number: SRR3716680). Transcripts in trichomes vs leaves was available only as a single data set, and fold change calculations were based on sequencing a single library of each type.

In the second, *S. divinorum* plants were sprayed with aqueous 1 mM MeJA containing 0.1% (*v*/*v*) Tween-20 (or a control solution containing only Tween-20). The total application volume was 10 mL per plant. Control and experimental plants were covered with plastic bags for 4 h after spraying. Young leaves of approximately the same size were collected for RNA preparation three weeks after treatments. Total RNA samples were prepared by Trizol (Sigma-Aldrich) and further purified by EZNA RNA purification kit (Omega Biotech), followed by evaluations of RNA integrity by Bio-analyzer (Agilent Technologies). Libraries of four biological replicates were prepared and sequenced on an Illumina NovaSeq 6000 with paired‐end reads of 100 bp at the McGill Genome Centre (Montreal, QC, Canada).

Reads from each library were trimmed using Trimmomatic [102] and mapped to the assembled *S. divinorum* genome presented here using STAR [103]. The resulting BAMs were post processed to sort reads by coordinate and add read groups using Samtools [104]. Differential gene expression analysis was conducted between MeJA-treated and control samples using the R package DESeq2 [105]. All code used for this analysis is provided in Supplementary Methods file 2.

To identify genes over-represented in glandular trichomes as candidates in SalA biosynthesis we analyzed a publicly available mRNA library sequenced with 454 GS FLX technology (SRA accession SRX1900686). We followed the same read trimming and mapping protocol outlined above. Exploratory expression analysis was conducted using the R package EdgeR [106] where differential expression can be characterized without replication using an exact test.

## Supplementary Information

Supplementary Material 1.

Supplementary Material 2.

Supplementary Material 3.

Supplementary Material 4.

Supplementary Material 5.

## Data Availability

All data supporting the conclusions of this article are included within the article and its Supplementary files and datasets. The S. divinorum genome sequence described herein is available at NCBI (https://www.ncbi.nlm.nih.gov/) under the BioProject PRJNA1104206. RNAseq data reported in this study is available under accession SRR3716680. Detailed methods, including sample code for performing major analyses are available in Supplementary Methods files 1 and 2.
