# Supplementary material for: A chromosome level reference genome of Diviner’s sage (Salvia divinorum) provides insight into salvinorin A biosynthesis
Source: BMC Plant Biol. 2024 Oct 1;24:914. doi: 10.1186/s12870-024-05633-0 (PMC11443658; doi:10.1186/s12870-024-05633-0)
Supplement: Supplementary file 1 — Supplementary Material 1. [file 12870_2024_5633_MOESM1_ESM.pdf]

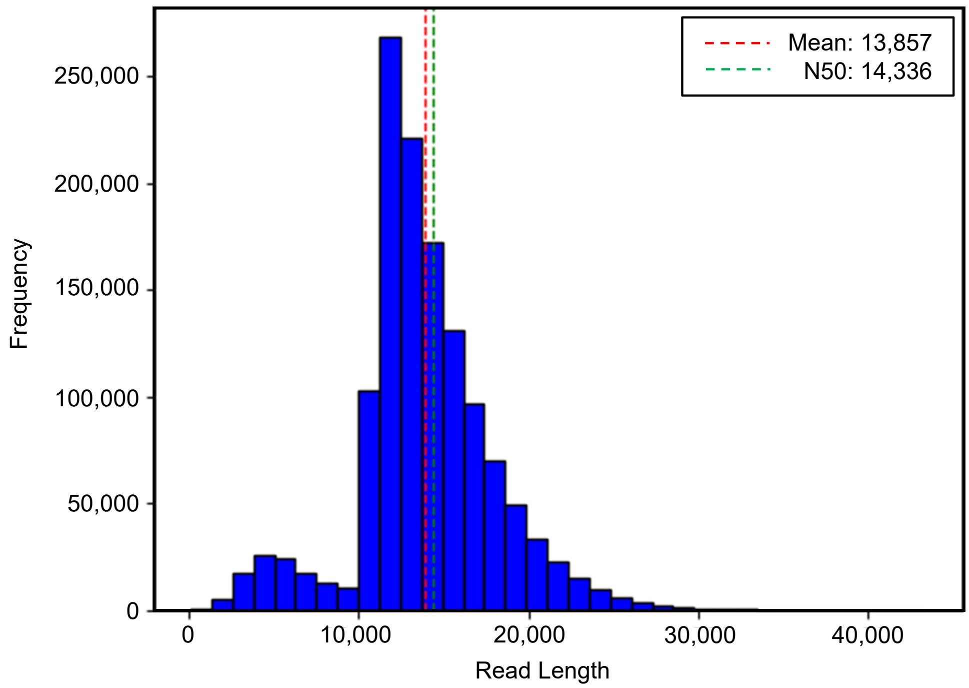

Supplementary Figure 1 Distribution of pacbio hifi read lengths used in the genome assembly described in this study**.**

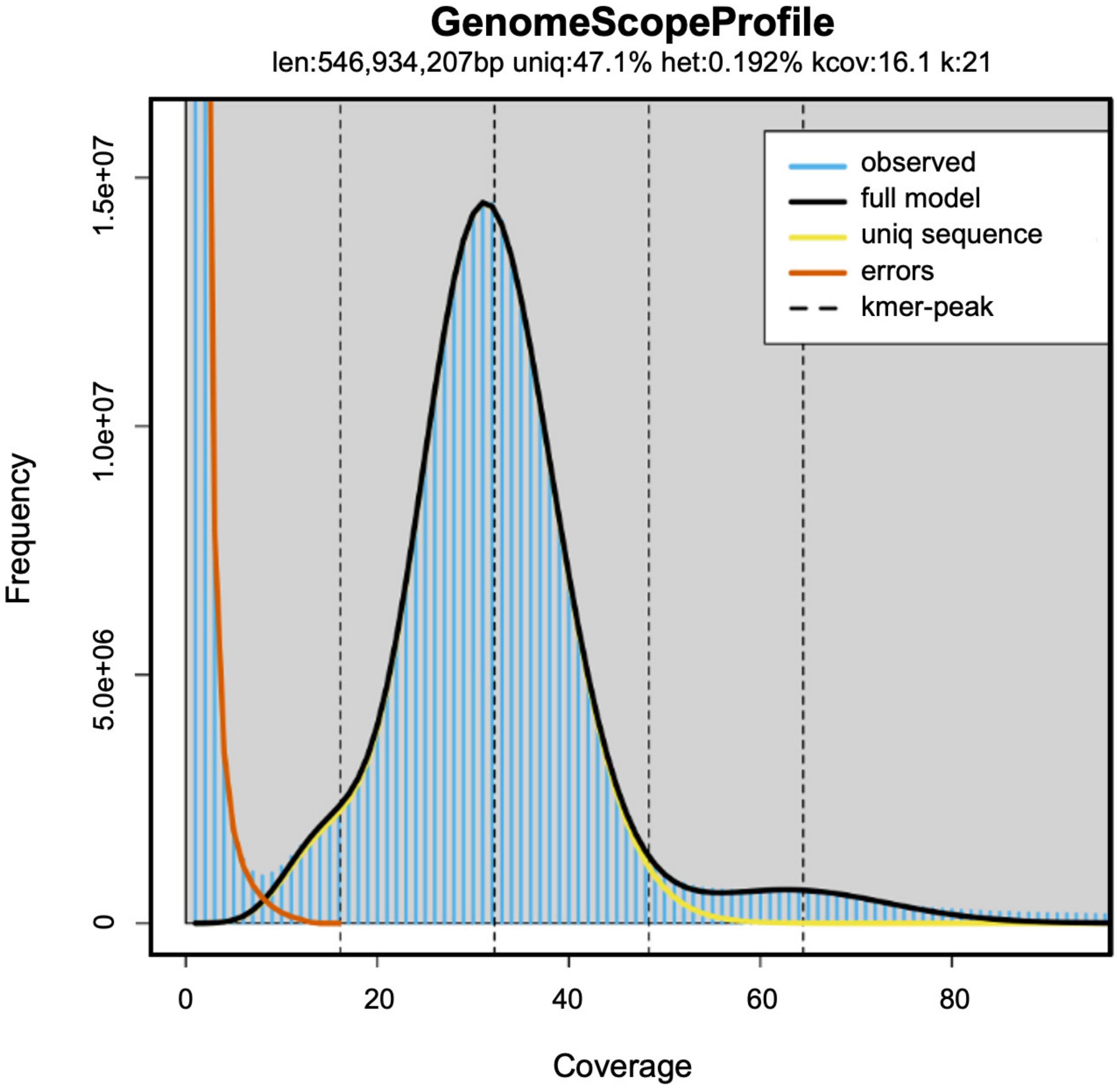

Supplementary Figure 2 Genome size, heterozygosity, repeat content and coverage estimation by GenomeScope using 21-mer counting

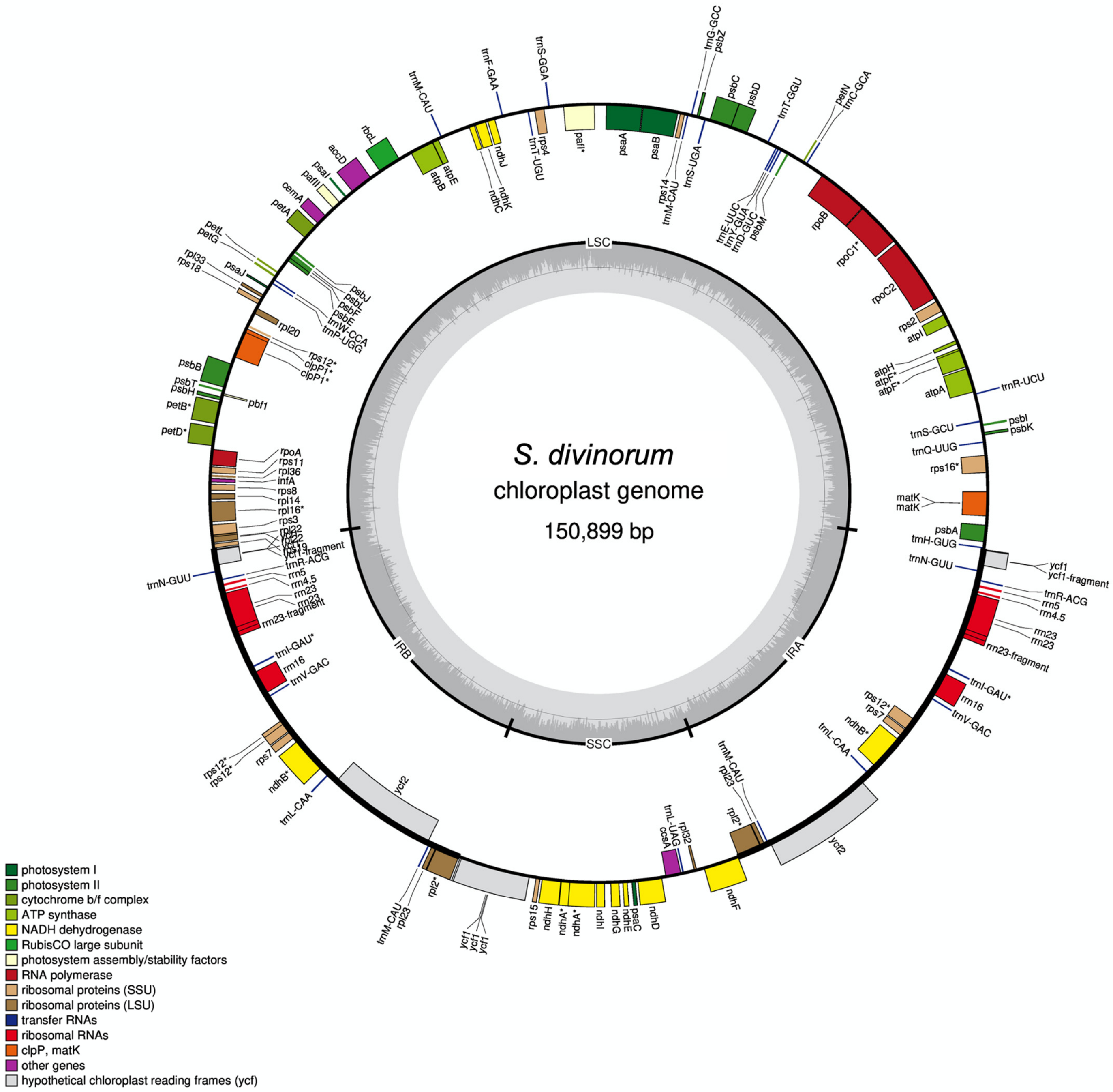

Supplementary Figure 3 *S. divinorum* chloroplast genome structure and gene annotations. Inner grey ring displays GC content.

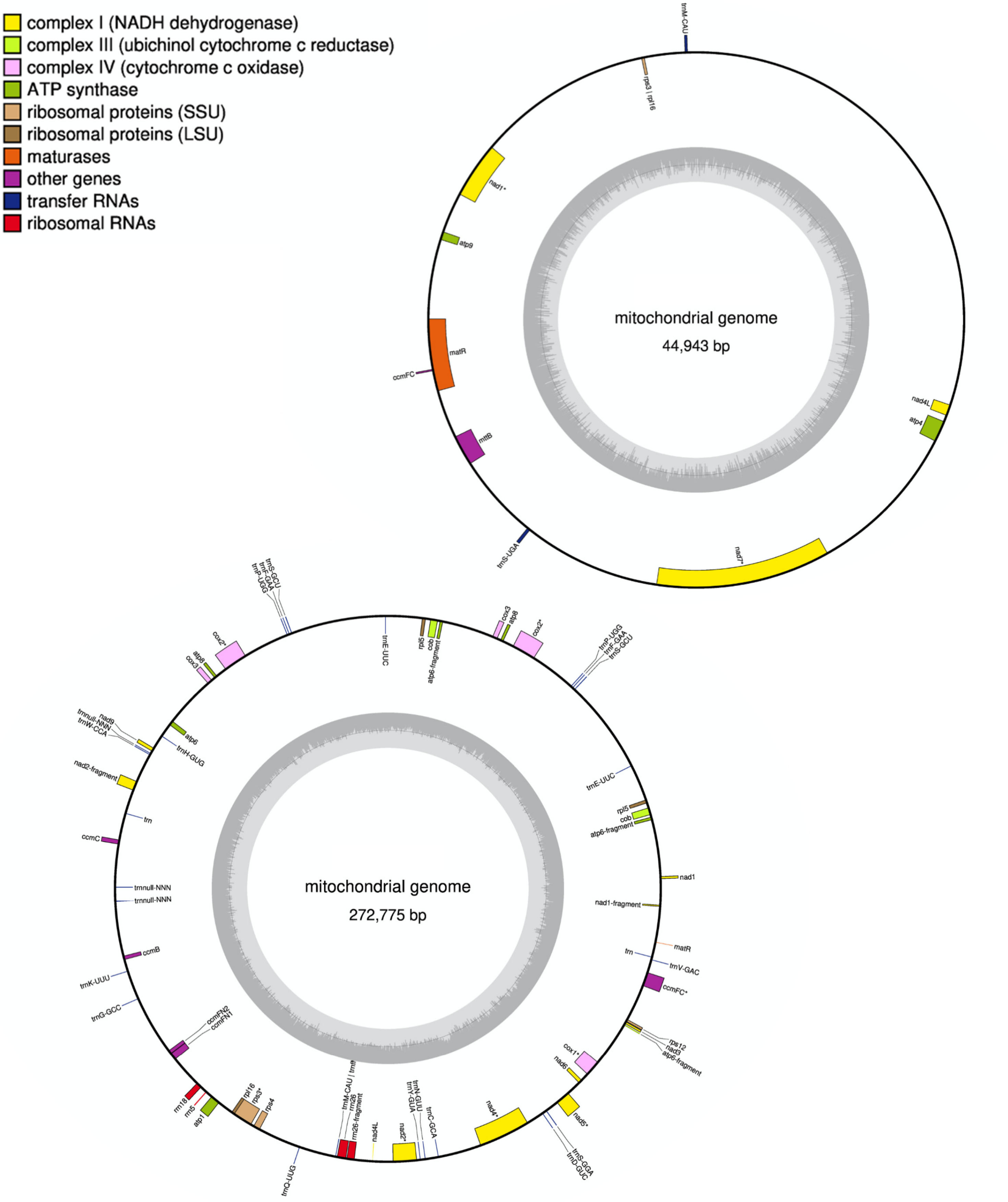

Supplementary Figure 4 *S. divinorum* mitochondrial genome structure and gene annotations. Inner grey ring displays GC content.

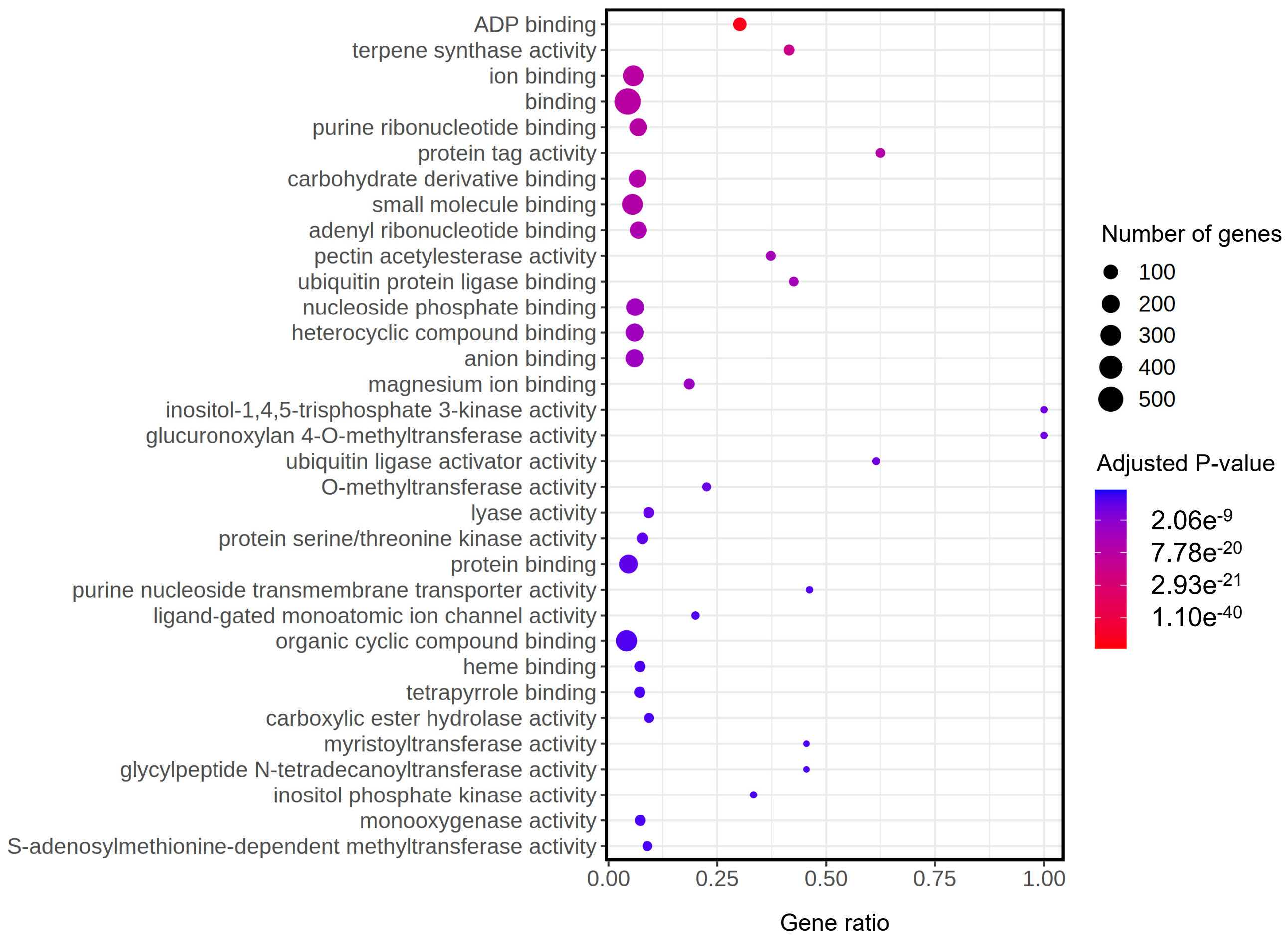

Supplementary Figure 5 Significantly ($p<0.05$) enriched GO molecular function annotations in expanded gene families of *S. divinorum*.

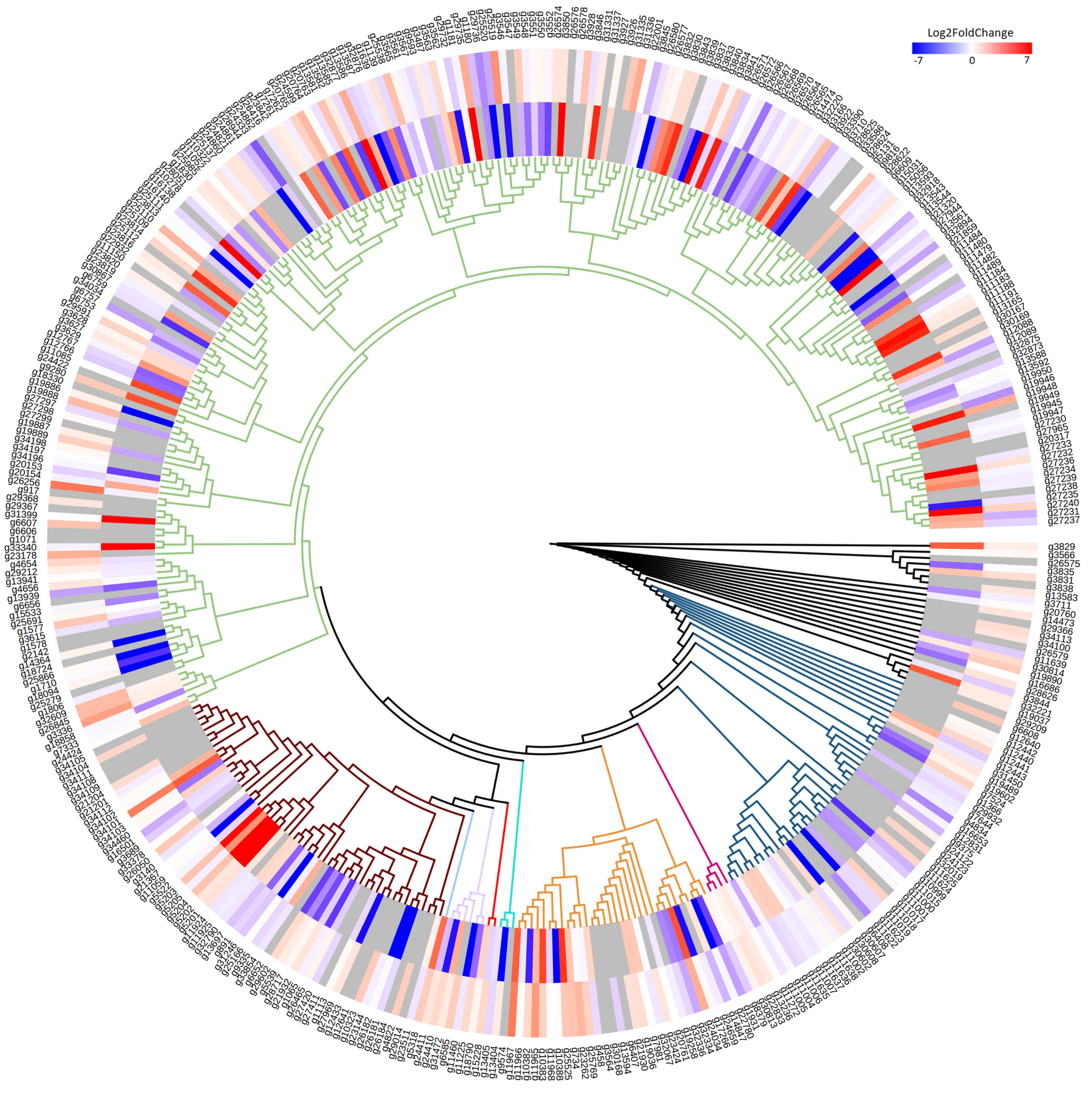

Supplementary Figure 6 Neighbor-joining tree of *S. divinorum* cytochrome P450 genes. Branch colors depict CYP clans (green, CYP71; brown, CYP85; light blue, CYP710; light purple, CYP74; red, CYP51; turquoise, CYP711; orange, CYP96; purple, CYP97; navy, CYP72; black, unclassified). Inner heatmap ring represents log_2 fold change of expression in trichomes compared to whole leaf; outer heatmap represents log_2fold change in expression in methyl jasmonate induced compared to control leaves.

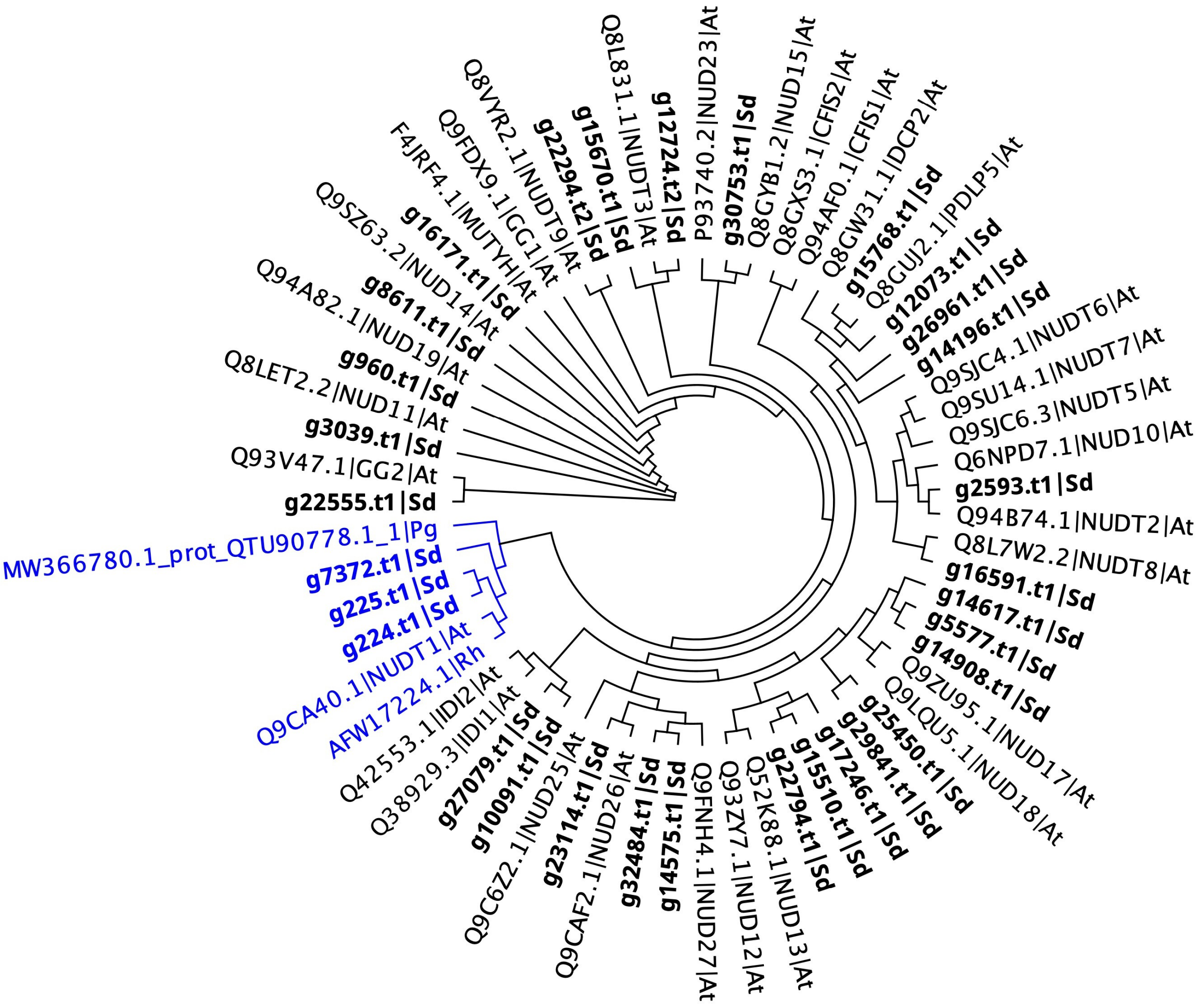

Supplementary Figure 7 Unrooted neighbor-joining phylogeny of Nudix hydrolases in Arabidopsis thaliana (At) and *S. divinorum* (Sd; bold). The terpene modifying clade of Nudix hydrolases (Bergmann et al., 2021) is shown in blue and includes sequences from *Rosa hybrida* (Rh) and *Pelargonium graveolens* (Pg) in addition to *S. divinorum* and *A. thaliana.*

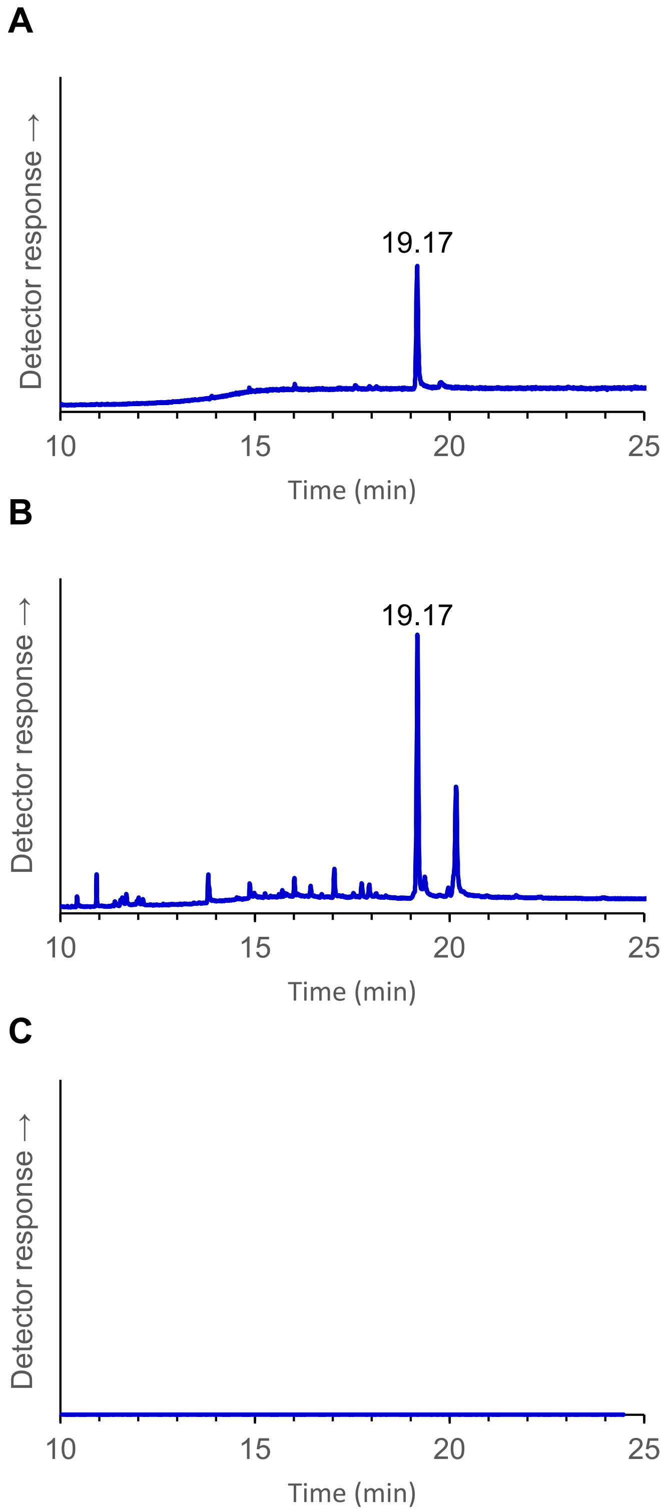

Supplementary Figure 8 Detection of salvinorin A in extracts of *Salvia divinorum* leaves. **A**, Gas chromatography – mass spectrometry analysis of a salvinorin A standard showing its elution at 19.17 min. **B**, Extract of *S. divinorum* leaf. **C**, Solvent only blank.

Supplementary Table 1. Differential expression of genes implicated in SalA biosynthesis

Gene name	Annotation numbers	Expression levels by Base Mean	Log_2 FC[a] (MeJa induction)	Adjusted *p*-value (MeJa induction)	Log_2 FC in trichome
SdCPS2	g10209	55	0.59	0.76	7.71
SdCS	g32877	2,991	2.95	0.02	7.15
SdC1H	g5201	1,809	1.01	0.59	8.08
SdC18H	g5204	1,130	2.17	0.01	7.07

[a] FC, fold change
