# Supplementary material for: A chromosome level reference genome of Diviner’s sage (Salvia divinorum) provides insight into salvinorin A biosynthesis
Source: BMC Plant Biol. 2024 Oct 1;24:914. doi: 10.1186/s12870-024-05633-0 (PMC11443658; doi:10.1186/s12870-024-05633-0)
Supplement: Supplementary file 4 — Supplementary Material 4. [file 12870_2024_5633_MOESM4_ESM.pdf]

Supplementary Methods file 1

A chromosome level reference genome of Diviner's sage (*Salvia divinorum*) provides insight into salvinorin A biosynthesis

Scott A. Ford, Rob W. Ness, Moonhyuk Kwon, Dae-Kyun Ro, Michael A. Phillips

Detailed Methods

Contents

Genome Assembly……………………………………………….....2
Organelle genome annotation……………………………….……….5
Structural annotation ………………………………...……....7
Functional annotation……………………………………………....9
Identification of diterpene biosynthetic gene clusters…………11
Genome evolution……………………………………………....11
Circos plotting…………………………………………………...19

A - Genome assembly

Text within [ ] represents the names of files that have been simplified for clarity.

1. Estimate genome size

Software: Jellyfish v2.2.10

```
jellyfish count <(zcat [reads.fasta.gz]) -m 21 -t 10 -s 800M -C
jellyfish histo -t 10 --high=100000 mer_counts.jf > reads.histo
```

Software: GenomeScope online tool http://genomescope.org/

```
Kmer length = 21
Read length = 14000
Max kmer coverage = 100000
```

2. Draft genome assembly

Software: hifiasm

```
hifiasm -o assembly.asm -t 32 [reads.fastq.gz] 2> assembly.log
```

3. Purge erroneous allelic duplication and low coverage contigs (https://github.com/dfguan/purge_dups)

i. Map HiFi reads to hifiasm assembly

Software: Minimap2 v2.22

```
minimap2 -x map-hifi [assembly] [reads.fastq.gz] > [asm.paf.gz]
```

ii. From alignment, get PB.base.cov, PB.stat files and caculate cutoffs for purge_dups

Software: purge_dups v1.2.5

```
pbcstat [asm.paf.gz] # produces PB.base.cov and PB.stat files
calcuts PB.stat > [cutoffs] 2>calcults.log # calculates cutoffs
```

iii. Split assembly; perform self-alignment

Software: purge_dups v1.2.5

```
split_fa [assembly] > [asm.split]
```

Software: Minimap2 v2.22

```
minimap2 -xasm5 -DP [asm.split] [asm.split] | gzip -c >
[asm.split.self.paf.gz]
```

Software: purge_dups v1.2.5

```
#command below is based on calculated cutoffs, but cutoffs can be
adjusted manually. See [calcults.log] to determine if manual
adjustment is required. See documentation for tutorial

purge_dups -2 -T [cutoffs] -c [PB.base.cov]
[asm.split.self.paf.gz] > [dups.bed] 2> [purge_dups.log]
```

iv. Get sequences back following after haplotigs are removed

Software: purge_dups v1.2.5

```
get_seqs -e [dups.bed] [assembly] > [asm.purged.fa] 2>
[asm.hap.fa] #haplotig fasta
```

4. Assemble organelle genomes

Software: ptGAUL

i. CpDNA

```
ptGAUL.sh -t 16 -r [relatedCpDNA.fasta] -l [reads.fastq.gz] -o
[chloro.out]
```

ii. MitoDNA

```
# map reads to mito genomes of related species with minimap2;
#filter for length > 1000 bp and residue matches/length >0.7;
extract names of reads

minimap2 -x map-hifi [relatedMtDNA] [reads.fastq.gz] >
[readsToMt.paf] 2>minimap_error.log \
&& awk '{print $1, $10, $11, $10/$11}' readsToMt.paf >
[filter1.paf] \
# get names of reads which map to mito
&& awk '{if (($4>=0.7) && ($3 >=1000)) {print $1}}'
[filter1.paf]> [filter_names.txt]

# get sequences of reads which map to mito
```

```
seqkit fq2fa [reads.fastq.gz] | grep -A 1 -Ff [filter_names.txt]
| grep -v "^--$" > [filter_sequences.fa]

# based on length of filter_sequences.fa , and predicted length
of mtDNA, calculate the factor by which you need to downsample
reads to get to ~60x coverage (see line below) – I am calling
this number 'f':
# mtGenomelength x 60 /length of reads = f

# install seqtk https://github.com/lh3/seqtk

seqtk sample -s100 [filter_sequences.fa] [f]  >
[filter_sequence_subsampled.fa]

# assemble subsampled reads using hifiasm to get mito organelles

hifiasm -o [mito.asm] -t 32 [filter_sequences_subsampled.fa
```

5. Filter redundant organellular DNA contigs from final assembly

```
# Map assembled contigs to the assembled plastid and mito genomes

minimap2 -c [cpDNA_mtDNA.fasta] [assembly.fa] > [asm
_cpDNA_mtDNA_aln.paf]

# Filter contigs which map to organelles at >99% of their length
awk -F'\t' '{
    col2 = $2;
    col3 = $3;
    col4 = $4;
    diff = (col3 > col4) ? col3 - col4 : col4 - col3;
    percent_col2 = (diff / col2) * 100;
    if (percent_col2 > 99) {
        print;
    }
}' [asm_cpDNA_mtDNA_aln.paf] >
[asm_cpDNA_mtDNA_aln.filtered.length.99.paf]

# python function to read PAF file
     def read_paf(paf_file):
          contigs_to_remove = set()
          with open(paf_file, 'r') as f:
               for line in f:
                    parts = line.split('\t')
                    contig_name = parts[0]
                    contigs_to_remove.add(contig_name)
          return contigs_to_remove
```

```
# and to remove cpDNA and mtDNA contigs from the assembly
     def filter_fasta(input_fasta, output_fasta,
contigs_to_remove):
          with open(input_fasta, 'r') as f_in,
open(output_fasta, 'w') as f_out:
               remove = False
               for line in f_in:
                    if line.startswith('>'):
                         contig_name = line.strip().lstrip('>')
                         if contig_name in contigs_to_remove:
                              remove = True
                         else:
                              remove = False
                    if not remove:
                         f_out.write(line)

# execute python functions to obtain filtered assembly

contigs_to_remove=read_paf(asm_cpDNA_mtDNA_aln.filtered.length.99
.paf)

filter_fasta(assembly.fa, assembly_filtered_CpMtDNA.fasta,
contigs_to_remove)
```

B – Organelle Genome Annotation

Functional annotation of organelle genomes was done using the webtool GeSeq (https://chlorobox.mpimp-golm.mpg.de/geseq.html), using the reference mitochondrial genome of *Arabidopsis thaliana*, and reference plastid genomes of *S. splendens*, *S. miltiorrhiza*, and *S. hispanica* (See Methods section). The following options were used.

For mtDNA annotation:
- BLAT - CDS, tRNA, rRNA
- ARGAGON - default
- tRNAscan – default

For cpDNA annotation:
- BLAT - CDS, tRNA, rRNA
- ARGAGON - default
- tRNAscan – default
- HMMER profile search
- Support annotation by Chloë
- Annotate plastid Inverted Repeat (IR)
- Annotate plastid trans-spliced *rps12*

C – Structural Annotation

1. Generate species-specific repeat library, combine with RepBase

Software: RepeatModeler v2.0.5

```
# Use singularity to install container for required software from
https://github.com/Dfam-consortium/TETools

singularity pull dfam-tetools-latest.sif
docker://dfam/tetools:latest

# Using the singularity container, you can run the tools
BuildDatabase, RepeatModeler, and RepeatMasker

BuildDatabase -name [species_db] [assembly.fa]
RepeatModeler -LTRStruct -quick -database [species_db]

# This will produce a repeat library for your species called
species_db-families.fa
# Recommended to concatenate this with a broader repeat library,
such all Viridiplantae from RepBase
https://www.girinst.org/server/RepBase/index.php
```

2. Mask repeats using repeat library

Software: RepeatMasker v4.1.6

```
RepeatMasker -lib [repeat_library.fasta] -pa 8 -dir rp0 -gff -e ncbi
-s [assembly.fa] – dir [outdir]

# this generates a repeat-masked genome assembly, assembly.fa.masked
- Use this for gene prediction.
```

3. Predict genes using RNAseq and protein evidence

Software: BRAKER v3.0.8

```
# Install BRAKER3 SIF file, use it to run BRAKER commands
singularity build braker3.sif docker://teambraker/braker3:latest
```

```
# We created a protein database consisting of all Viridiplantae
proteins from OrthoDB v11 merged with Lamiaceae proteins downloaded
from UniProt (20240220) and called it
viridiplantae_lamia_merged.fasta


# First run BRAKER in protein-only mode
braker.pl –genome= [assembly.fa.masked] --gff3 --threads=32 --
prot_seq=[viridiplantae_lamia_merged.renamed.filtered.fasta]

# Repeat BRAKER annotation, but use only RNA
# Download raw reads and keep them in folder ‘rna’
braker.pl –genome= [assembly.fa.masked] --gff3 --threads=32 --
rnaseq_sets_dirs=rna/ -- rnaseq_sets_ids=[comma-separated list names
of RNAseq file names]
```

4. Predict genes using RNAseq and protein evidence

Software: TSEBRA (access from BRAKER3 SIF file)

```
# From BRAKER, you obtain gtf files and gff hinstfiles for each mode
(RNA and protein)
# We call them braker.RNA.gtf, braker.RNA.hintsfile.gff,
braker.Prot.gtf and braker.Prot.gff
# To use tsebra you must set up a config file ‘tsebra.cfg’ for
determining how each hintfile is weighted. See
https://github.com/Gaius-Augustus/TSEBRA for details.
# We used the below parameters in tsebra.cfg:
#P 0.5
#E 20
#C 0.5
#M 1
#intron_support 0.3
#stasto_support 1
#e_1 0.0
#e_2 0.5
#e_3 0.096
#e_4 0.02
#e_5 0.18
#e_6 0.18


# Run TSEBRA with following parameters
tsebra.py -g braker.RNA.gtf,braker.Prot.gtf \
        -c tsebra.cfg \
        -e braker.RNA.hintsfile.gff,braker.Prot.hintsfile.gff \
        --filter_single_exon_genes \
        -o tsebra.gtf 2> tsebra_purge_single-exons.log
```

5. Format TSEBRA output

The following is based on the pipeline by J. Santangelo (https://github.com/James-S-Santangelo/dcg/tree/main)

```
# Remove organelles

# Define the organelle names as variables
mtDNA=[name of mtDNA contig]
cpDNA=[name of cpDNA contig]
awk -v org1="$mtDNA" -v org2="$cpDNA" '$1 != org1 && $1 != org2'
tsebra.gtf > tsebra.no-orgs.gtf

# Remove features other than CDS

awk '$3 != "exon" && $3 != "intron" && $3 != "gene" && $3 !=
"transcript"' tsebra.no-orgs.gtf > tsebra.no-orgs.CDS.gtf
```

Software: genometools

```
# convert to gff using AGAT v1.0.0-pl5321hdfd78af_0
(https://zenodo.org/records/5834795)

agat_convert_sp_gxf2gxf.pl --gtf tsebra.no-orgs.CDS.gtf \
    --output tsebra.no-orgs.CDS.gff &> agat_convert.log
```

Software: genometools

```
# Install genometools https://genometools.org/tools.html

gt gff3 -sort -tidy -retainids tsebra.no-orgs.CDS.gff > tsebra.no-
orgs.CDS.sorted.gff 2> gt_sort.log



# Fix transcript ID using python

input_file = 'tsebra.no-orgs.CDS.sorted.gff'
output_file = 'tsebra.no-orgs.CDS.sorted.fixed.gff'

with open(input_file, 'r') as fin:
    with open(output_file, 'w') as fout:
        lines = fin.readlines()
        for line in lines:
            if not line.startswith('#'):
                sline = line.split('\t')
                feature = sline[2]
                if feature == 'gene':
                    sline[8] = re.sub(r'(;transcript_id.*$)', '',
sline[8])
```

```
                elif feature == 'mRNA':
                    id_pattern = r"(?<=ID=)(.*)(?=;Parent)"
                    ID = re.search(id_pattern, sline[8]).group(1)
                    if ID.endswith('.t1'):
                        pass
                    else:
                        sline[8] = 
re.sub(r'(?<=;transcript_id=)(.*$)', ID, sline[8])
                fout.write('\t'.join(sline))
            else:
                fout.write(line)
```

```
# Get protein sequences using gffread from BRAKER SIF file

gffread -E -y proteins.fa -g assembly.fa tsebra.no-
orgs.CDS.sorted.fixed.gff 2> gffread.log
```

D – Functional Annotation

We combined functional annotations from eggNOG-Mapper, InterProScan and funannotate.

Software: eggNOG-Mapper

```
# Get eggNOG-Mapper (https://github.com/eggnogdb/eggnog-mapper). We
used conda for installation.


download_eggnog_data.py -y --data_dir db


# Use proteins from previous step for input
emapper.py -i [proteins.fa] \
    --data_dir db \
    --itype proteins \
    -o [proteins.eggNOG.out] \
&> eggnog.log

# output .emapper.annotations is used as input for funannotate
(below)
```

Software: InterProScan

```
# Install version 5.66 as below.
#Set versions
version_major=5.66
version_minor=98.0
```

```
wget
http://ftp.ebi.ac.uk/pub/software/unix/iprscan/5/${version_major}-
${version_minor}/interproscan-${version_major}-${version_minor}-64-
bit.tar.gz.md5

wget
http://ftp.ebi.ac.uk/pub/software/unix/iprscan/5/${version_major}-
${version_minor}/interproscan-${version_major}-${version_minor}-64-
bit.tar.gz

tar -xvzf interproscan-${version_major}-${version_minor}-64-
bit.tar.gz

python3 setup.py -f interproscan.properties


interproscan-5.66-98.0/interproscan.sh -i [proteins.fa] -d
interproscan/out/ -appl
CDD,FUNFAM,Gene3D,HAMAP,ANTIFAM,PANTHER,Pfam,PIRSF,PIRSR,PRINTS,SFLD
,SMART,SUPERFAMILY,NCBIFAM --goterms --pathways

# .xml output file is used as input for funannotate (below)
```

Software: Funannotate

```
# combines annotations and queries additional DBs

# Install with singularity
singularity pull docker://nextgenusfs/funannotate

funannotate setup -d db -b embryophyta --force 2> setup.errors


funannotate annotate --gff tsebra.no-orgs.CDS.sorted.fixed.gff
--fasta assembly.fa.masked –iprscan [proteins.xml] --eggnog
[proteins.emapper.annotations] --force --cpus 32 --out [out_dir]
--busco_db embryophyta

# output directory contains proteins, transcripts, gff, annotation
tables, stats
```

The gff file from funannotate is annotated with pfam and InterPro domains which were used to identify enzymes in classes involved to SalA synthesis (See Methods; Supplementary Data Set 1L for details). As described in the Methods, protein-protein BLAST searches against characterized proteins from closely related species were used to identify diterpene synthases. CYPs were putatively classified into clans and subfamilies similarly using a protein-protein BLAST search of predicted *S. divinorum* (based on pfam and InterPro domains) CYPs against a database of 9739 plant CYPs previously classified by Dr. David Nelson (https://drnelson.uthsc.edu/plants) (See Extended Data Set 2).

Based on the pipeline provided by J. Santangelo (https://github.com/James-S-Santangelo/dcg), if a protein was annotated as 'hypothetical protein', but was assigned a fully resolved enzyme commission (EC) number, the annotation was replaced with the EC number's product in the ExPASSY Enzyme database.

E – Identification of diterpene biosynthetic gene clusters (BGCs)

The PlantiSMASH (https://github.com/plantismash/plantismash) webserver was used with default settings to detect signatures of BGCs. Terpene synthesis clusters were further classified as diterpene clusters based on overlap with predicted diterpene synthase genes (See above).

F – Genome evolution

1. Pairwise synteny analysis with GENESPACE

The protein fasta file and gff file for *S. splendens* was downloaded from NCBI (Supplementary Data Set 1L for accession number). It needs to be converted to a bed file for GENESPACE analysis.

```
#first, python code to convert NCBI gff file to bed file suitable
for genespace

# Read the GFF file into a DataFrame
gff_file = 'Salvia_splendens.gff'
gff_df = pd.read_csv(gff_file, sep='\t', comment='#', header=None)

# Set column names
gff_df.columns = ['chrom', 'source', 'feature', 'start', 'end',
'score', 'strand', 'frame', 'attributes']

# Filter for only 'gene' entries
genes_df = gff_df[gff_df['feature'] == 'gene']

# Extract necessary columns and format as BED
bed_df = genes_df[['chrom', 'start', 'end', 'attributes']].copy()
bed_df['attributes'] = bed_df['attributes'].apply(lambda x:
x.split('Name=')[1].split(';')[0] if 'Name=' in x else '.')

# Write to BED file
bed_file = 'Salvia_splendens.bed'
bed_df.to_csv(bed_file, sep='\t', header=False, index=False,
columns=['chrom', 'start', 'end', 'attributes'])
```

The protein fasta file header must exactly match the bed file. The following python code accomplishes this, ensuring only the longest isoform is retained.

```
def extract_longest_isoform(fasta_file, output_file):
    sequences = {}

    # Pattern to extract SASPL_##### from the header
    pattern = re.compile(r'SASPL_\d+')

    # Read the input FASTA file
    for record in SeqIO.parse(fasta_file, "fasta"):
        match = pattern.search(record.description)
        if match:
            identifier = match.group(0)

            # Update the dictionary with the longest isoform
            if identifier not in sequences or len(record.seq) >
len(sequences[identifier].seq):
                sequences[identifier] = record

# Write the results to the FASTA file with >SASPL_####### as header
to match bed
    with open(output_file, "w") as out_file:
        for seq_id, record in sequences.items():
            out_file.write(f">{seq_id}\n")
            out_file.write(f"{record.seq}\n")

# Define input and output file names
input_fasta = "Salvia_splendens_SspV2_proteins.faa"
output_fasta = "Salvia_splendens.faa"

extract_longest_isoform(input_fasta, output_fasta)
```

S. divinorum bed and corresponding protein fasta filtes are similarly obtained, starting with output from functional annotation. The ID column from the bed file exactly matches the fasta header. The files are called `S_divinorum.bed` and `S_divinorum.faa`.

To set up genespace directory, create a folder called bed and add `S_divinorum.bed` and `S_splendens.bed`. Create another folder called peptide and add `S_divinorum.faa` and `S_splendens.faa`.

Software: GENESPACE

```
# Install GENESPACE(https://github.com/jtlovell/GENESPACE) using
conda. Verify a valid installation of Orthofinder and R
# Download MCScanX (follow instructions at
https://github.com/wyp1125/MCScanX)
```

```
unzip MCscanx.zip
cd MCScanx
make

# Execute GENESPACE run in R

library(GENESPACE)
wd <- getwd() # make sure you are the directory with peptide and bed
folders
path2mcscanx <- "[PATH/TO/MCScanx]"

gpar <- init_genespace(
  wd = wd,
  genomeIDs = c("S_splendens", "S_divinorum"),
  ploidy = c(2, 1), # S. splendens is tetraploid
  path2mcscanx = path2mcscanx,
  nCores = 24)# verify this runs without warning

run_genespace(gpar)


# Riparian plot can be customized following the run to change
labels, chromosome orders, colors, number of chromosomes plotted etc
# Tutorial available here:
https://htmlpreview.github.io/?https://github.com/jtlovell/tutorials
/blob/main/riparianGuide.html
```

2. Find orthogroups and orthologs

Protein sequences (.faa) for *S. hispanica*, *S. bowleyana*, *S. miltiorrhiza*, *S. rosmarinus* and *Sesamum indicum* were downloaded from NCBI (Supplementary Data Set 1L for accession numbers). Fasta files were filtered to retain only the longest isoforms of each protein. Due to differences in the formatting of fasta headers and corresponding gff files, proteins of each species were filtered separately using in-house python scripts. Along with the longest protein isoforms of *S. divinorum* and *S. splendens* (above), they were placed in a folder we are calling `comparative_genomes` for simplicity. Ensure that each fasta file has a species-specific identifier in the header, as this will be important for downstream analyses of gene families.

Software: OrthoFinder

```
# Install OrthoFinder (https://github.com/davidemms/OrthoFinder) or
use GENESPACE conda environment which includes orthofinder

# comparative_genomes is the directory with protein fastas
orthofinder -t 32 -a 32 -M msa -S diamond -A mafft -T fasttree -f
[comparative_genomes]

# An output folder called Species_Tree will contain a tree file
called SpeciesTree_rooted.txt
```

```
# For downstream analysis of gene family expansions and
contractions, the species tree needs to be made ultrametric
# OrthoFinder provides a python script for this conversion. You must
provide a root divergence time after the -r flag. We obtained this
from previous studies (See Methods)

make_ultrametric.py -r 55 SpeciesTree_rooted.txt

# the output ultrametric tree file is called
SpeciesTree_rooted.txt.ultrametric.tre
```

3. Gene family expansion and contraction

Gene family expansion and contraction analysis was done using CAFE5 (https://github.com/hahnlab/CAFE5), based on the ultrametric tree file `SpeciesTree_rooted.txt.ultrametric.tre` and orthogroup gene counts obtained from OrthoFiner (above). Install CAFE5 according to instructions provided (https://github.com/hahnlab/CAFE5).

Following OrthoFinder run, an Orthgroup gene count file `Orthogroups.GeneCount.tsv` will be in a directory called `Orthogroups`. This file must be slightly reformatted for use with CAFE5. Code for prepping `Orthogroups.GeneCount.tsv` is provided below based on a previously described pipeline (https://github.com/elsemikk/Willisornis_Genome_Assembly/blob/master/4.2_Gene_Family_Expansions.md). A detailed tutorial for CAFE5 is also available https://iu.app.box.com/v/cafetutorial-files .

Software: CAFE5

```
# First, remove the last column 'Total' which is not needed for
CAFE, then add a null first column
awk 'BEGIN{OFS="\t"}NF{NF-=1};1' Orthogroups.GeneCount.tsv | awk
'BEGIN{OFS="\t"}{print "(null)\t"$0}' > Cafe_input.tsv

# Temporarily remove the headers, which will allow you to run the
CAFE python script called clade_and_size_filter.py. This removes
orthogroups in which a single species has > 100 gene copies.

tail -n +2 Cafe_input.tsv > temp_filterinput

# Then, filter orthogroups using clade_and_size_filter.py

python path/to/CAFE5/docs/tutorial/clade_and_size_filter.py -i
temp_filterinput -o filtered_Cafe_input.tsv -s

# Put the header back and deal with the removal of the first
orthogroup by clade_and_size_filter.py

grep OG0000000 filtered_Cafe_input.tsv > temp_OG0
```

```
grep -v OG0000000 filtered_Cafe_input.tsv > temp_smallfams
grep -v OG0000000 large_filtered_Cafe_input.tsv > temp_largefams
head -n 1 Cafe_input.tsv > temp_header

# Manually check temp_OGO to see if a single species has >100 gene
copies. In our case it did so, code below is to add it to
temp_largefams, which will not be used for CAFE5 analysis
# If it does not, it can be used in analysis, so add it to
temp_smallfams instead

cat temp_header temp_smallfams > filtered_Cafe_input.tsv
cat temp_header temp_OG0 temp_largefams >
large_filtered_Cafe_input.tsv # not used for analysis
rm temp_OG0 temp_smallfams temp_largefams temp_header
temp_filterinput

# filtered_Cafe_input.tsv is used for analysis with CAFÉ
```

An initial run of CAFE5 is used to estimate the global error model file. Inputs are `filtered_Cafe_input.tsv` and `SpeciesTree_rooted.txt.ultrametric.tre` from previous two steps.

```
cafe5 -i filtered_Cafe_input.tsv -t
SpeciesTree_rooted.txt.ultrametric.tre --cores 32 -e -o
cafe_out/error_model > error_model.log

#error model is now in: cafe_out/error_model/Base_error_model.txt
```

Rerun CAFE5, this time using predicted error model.

```
cafe5 -i filtered_Cafe_input.tsv -t
SpeciesTree_rooted.txt.ultrametric.tre --cores 32 -
ecafe_out/error_model/Base_error_model.txt -o cafe_out/
single_lambda > single_lambda.log
```

Expansions and contractions for each branch of the tree (Figure 4) are provided in `cafe_out/single_lambda/Base_clade_results.txt.` Other output files in `cafe_out/single_lambda` are parsed to determine significantly expanded and contracted gene families in *S. divinorum* as shown below.

```
# Note that Salvia divinorum is labeled Salvia_divinorum<5> in
CAFE outputs

# First obtain list of significantly expanded and contracted
orthogroups in this species
grep "Salvia_divinorum<5>\*" Base_asr.tre |awk '{print $2}' >
S_divinorum/S_divinorum_sig_changes

# Then get list of all expanded orthogroups
```

```
awk '$5 > 0 {print $1,$3}' Base_change.tab >
S_divinorum/S_divinorum_all_expansions

# And all contractions
awk '$5 < 0 {print $1,$3}' Base_change.tab >
S_divinorum/S_divinorum_all_contractions

# Intersect lists to obtain significantly expanded orthogroups
grep -F -f S_divinorum/S_divinorum_sig_changes S_divinorum/
S_divinorum_all_expansions | awk '{print $1}' > S_divinorum/
S_divinorum_sig_expansions

# And significantly contracted orthogroups
grep -F -f S_divinorum/S_divinorum_sig_changes S_divinorum/
S_divinorum_all_contractions | awk '{print $1}' > S_divinorum/
S_divinorum_sig_contractions
```

4. GO enrichment analysis of expanded gene families

We next extract fasta files of protein sequences in significantly expanded gene families of *S. divinorm*, from the OrthoFinder output folder `Orthogroup_Sequences`, using the bash command below. Once this is performed, orthogroup sequences are placed in a folder `expanded_OGs`

```
while read Orthogroup ; do mv
[/path/to/Orthogroup_Sequences/"$Orthogroup".fa]
[path/to/expanded_OGs] ; done < S_divinorum_sig_expansions # from
above
```

Each fasta file in `expanded_OGs` represents an expanded gene family of *S. divinorum*, but contains sequences from multiple species, so we need to extract the names of sequences corresponding specifically to *S. divinorum*. The headers of *S. divinorum* sequences have a unique locus tag `AAHA92`, which is used for extracting the names of genes in expanded orthogroups of *S. divinorum*.

```
# First, within expanded_OGs directory, concatenate all expanded
OG fastas into one

cat *.fa > Sd_expanded_Ogs_all_sp.fasta

# Awk command to extract S_divinorum headers to a list

awk '/^>AAHA92_/ {gsub(/^>/, "", $0); print $1}'
Sd_expanded_Ogs_all_sp.fasta > Sd_expanded_OG_gene_names.txt
```

This provides a list of the names for proteins within significantly expanded gene families of *S. divinorum*. It is called `Sd_expanded_OG_gene_names.txt`, and is used as input for GO enrichment analysis using GOATOOLS (https://github.com/tanghaibao/goatools).

In addition to this list, GOATOOLS requires as input, a list of:

i. All gene names in the genome which have GO terms assigned, formatted as shown below.

```
AAHA92_g121.t1
AAHA92_g122.t1
AAHA92_g124.t1
AAHA92_g126.t1
AAHA92_g127.t1
AAHA92_g128.t2
```

ii. GO terms for each gene in the genome formatted as shown below:

```
AAHA92_g121.t1   GO:0005634;GO:0006355
AAHA92_g122.t1   GO:0061630
AAHA92_g124.t1   GO:0005515;GO:0002098;GO:0033588
AAHA92_g126.t1   GO:0005515
AAHA92_g127.t1   GO:0006644;GO:0008195
AAHA92_g128.t2   GO:0005313;GO:0022857;GO:0015185
```

iii. All gene names in expanded gene families which have GO terms assigned, formatted as shown below.

```
AAHA92_g224.t1
AAHA92_g845.t1
AAHA92_g846.t1
AAHA92_g1001.t1
AAHA92_g1007.t1
AAHA92_g1010.t2
```

To generate these input files, we first extract names from the fasta file which contains longest isoform of each gene. We call this fasta file `longest_isoforms.faa` for simplicity.

```
awk '/^>AAHA92_/ {gsub(/^>/, "", $0); print $1}'
longest_isoforms.faa > [Sd_longestIso_gene_IDs.txt]
```

We then use the python code below to identify genes in `Sd_longestIso_gene_IDs.txt` that have assigned GO terms during functional annotation, and extract them to a line separated list. As input, we use the funannotate-generated gff file, which for simplicity we call `funannotate_out.gff`. As output we obtain the required list of genes which have GO terms assigned, and their corresponding GO terms formatted for input to GOATOOLS. We call this output file `all_genes_GO_terms.txt` as shown below.

```
import pandas as pd

# File paths
```

```
gff_file_path = path/to/funannotate_out.gff
gene_ids_file_path = path/to/Sd_longestIso_gene_IDs.txt
output_file_path = 'all_genes_GO_terms.txt'

# Load gene IDs from a text file into a set for fast access
with open(gene_ids_file_path, 'r') as file:
    gene_ids = {line.strip() for line in file}

# Read the GFF file
cols = ['seqname', 'source', 'feature', 'start', 'end', 'score',
'strand', 'frame', 'attribute']
gff_data = pd.read_csv(gff_file_path, sep='\t', names=cols,
comment='#')

# Filter mRNA entries
mRNA_data = gff_data[gff_data['feature'] == 'mRNA']

# Extract GO terms and gene ID
def extract_go_terms(attributes):
    parts = {x.split('=')[0]: x.split('=')[1] for x in
attributes.split(';') if '=' in x}
    go_terms = parts.get('Ontology_term', '')
    return go_terms.replace(',', ';')

# Create a new DataFrame for results
results = []

# Iterate over mRNA data
for _, row in mRNA_data.iterrows():
    attributes = row['attribute']
    gene_id = attributes.split(';')[0].split('=')[1]

    if gene_id in gene_ids:
        go_terms = extract_go_terms(attributes)
        if go_terms:
            results.append((gene_id, go_terms))

# Save results to a file
with open(output_file_path, 'w') as f:
    for gene_id, go_terms in results:
        f.write(f"{gene_id}\t{go_terms}\n")
```

We then extract the first column of `all_genes_GO_terms.txt` to a separate list of gene IDs only, which we call `all_genes_GO_namesOnly.txt`. This file is used as input *i* for GOATOOLS.

```
awk '{print $1}' all_genes_GO_terms.txt >
all_genes_GO_namesOnly.txt
```

Finally, to generate input *iii* we filter `Sd_expanded_OG_gene_names.txt` such that it includes only genes annotated with GO terms. This can be done in python as below. We call the output `Sd_expanded_OG_gene_names_filtered.txt`

```
# Load the list of gene IDs in expanded OGs
with open('Sd_expanded_OG_gene_names.txt', 'r') as file:
    exp_gene_IDs = set(line.strip() for line in file.readlines())

# Load in list of all gene IDs which have GO terms
with open('all_genes_GO_namesOnly.txt', 'r') as file:
    all_gene_IDs = set(line.strip() for line in file.readlines())

# Find the intersection of both sets
common_genes = exp_gene_IDs.intersection(all_gene_IDs)

# Write the common gene IDs to a new file
with open('Sd_expanded_OG_gene_names_filtered.txt', 'w') as file:
     for gene_id in common_genes: file.write(f"{gene_id}\n")
```

Now we have all required inputs for GOATOOLs. For instructions on installation, see (https://github.com/tanghaibao/goatools).

Software: GOATOOLS

```
# Download .obo file of most current GO terms
wget http://current.geneontology.org/ontology/go-basic.obo


# Find enriched GO terms using find_enrichment.py script from
GOATOOLS

find_enrichment.py --obo go-basic.obo --pval=0.05
Sd_expanded_OG_gene_names_filtered all_genes_GO_namesOnly.txt
all_genes_GO_terms.txt --outfile [enriched_GO_terms]
```

Output from GOATOOLs is used for plotting in Figure 4b and Supplemental Figure 5 after redundant GO terms are filtered using REVIGO (http://revigo.irb.hr/).

G - CIRCOS plotting

Figure 5 was generated using CIRCOS (https://circos.ca/). The command to generate the plot is below:

```
circos -conf [configuration.conf] -png -nosvg -param
max_points_per_track=50000 -outputfile [outfile]
```

The configuration file `[configuration.conf]` is a text file with the following content:

```
karyotype = ../karyotype_info.txt


chromosomes_units = 1000000
chromosomes_reverse = ptg000005l_1

#### IDEOGRAM ####

<ideogram>

    <spacing>
        default = 0.01r
      <pairwise ptg000010l_1 ptg000001l_1>
           spacing = 0.25r
      </pairwise>

          <pairwise ptg000012l_1 ptg000005l_1>
               spacing = 0.25r
          </pairwise>

              <pairwise ptg000013l_1 ptg000006l_1>
                   spacing = 0.25r
              </pairwise>

                  <pairwise ptg000015l_1 ptg000013l_1>
                       spacing = 0.25r
                  </pairwise>

    </spacing>

    # Ideogram position, fill and outline
    radius           = 0.9r
    thickness        = 60p
    fill             = yes
    stroke_color     = black
    stroke_thickness = 2p

    # Minimum definition for ideogram labels.

    show_label       = yes
    # see etc/fonts.conf for list of font names
    label_font       = bold
    label_radius     = dims(ideogram,radius_inner) + 170p
    label_size       = 40
    label_case       = upper
    label_parallel   = yes

</ideogram>
```

```
#### TICKS ####

show_ticks          = yes
show_tick_labels    = yes

<ticks>
    radius           = 1r
    color            = black
    thickness        = 2p

    # the tick label is derived by multiplying the tick position
    # by 'multiplier' and casting it in 'format':
    #
    # sprintf(format,position*multiplier)
    #

    multiplier       = 1e-6

    # %d   - integer
    # %f   - float
    # %.1f - float with one decimal
    # %.2f - float with two decimals
    #
    # for other formats, see
http://perldoc.perl.org/functions/sprintf.html

    format           = %.1f

    <tick>
        spacing        = 10u
        size           = 20p
        show_label     = yes
        label_size     = 20p
        label_offset   = 5p
    </tick>
</ticks>

#### PLOTS ####

track_width = 0.05
track_pad   = 0.03
track_start = 0.97

<plots>

    ## GC CONTENT ##
    <plot>

        show  = yes
        type  = line
```

```
        file  = ../gc_content.txt
        r1  = eval(sprintf("%fr",conf(track_start)-
counter(plot)*(conf(track_width)+conf(track_pad))))
        r0  = eval(sprintf("%fr",conf(track_start)-
counter(plot)*(conf(track_width)+conf(track_pad))-
conf(track_width)))
        max   = 0.45
        min   = 0.20
        color = mygreen
        thickness = 4

        <backgrounds>
            <background>
                color     = greens-9-seq-1
                y1        = 1r
                y0        = 0r
            </background>
        </backgrounds>

        <rules>
            <rule>
                condition  = var(value) > 0.5
                show       = no
            </rule>
        </rules>

        <axes>
            <axis>
                color     = lgreen
                thickness = 1
                spacing   = 0.2r
            </axis>
        </axes>

    </plot>

    ## GENE DENSITY ##

    <plot>

        show  = yes
        type  = heatmap

        file  = ../gene_counts.txt
        r1  = eval(sprintf("%fr",conf(track_start)-
counter(plot)*(conf(track_width)+conf(track_pad))))
        r0  = eval(sprintf("%fr",conf(track_start)-
counter(plot)*(conf(track_width)+conf(track_pad))-
conf(track_width)))
        color_mapping = 2
        min = 0
        max = 80
```

```
        color = white,reds-9-seq,mydred

    </plot>
    ## REPEAT CONTENT ##
    <plot>

        show  = yes
        type  = heatmap

        file  = ../repeat_content.txt
        r1  = eval(sprintf("%fr",conf(track_start)-
counter(plot)*(conf(track_width)+conf(track_pad))))
        r0  = eval(sprintf("%fr",conf(track_start)-
counter(plot)*(conf(track_width)+conf(track_pad))-
conf(track_width)))
        color_mapping = 2
        min = 0
        max = 0.99999
        color = white,blues-9-seq,mydblue

    </plot>

    ## CYP CLUSTERS ##

    <plot>
        show  = yes
        type  = heatmap

        file = ../CYP_regions.txt
        r1  = eval(sprintf("%fr",conf(track_start)-
counter(plot)*(conf(track_width)+conf(track_pad))))
        r0  = eval(sprintf("%fr",conf(track_start)-
counter(plot)*(conf(track_width)+conf(track_pad))-
conf(track_width)))
        min  = 0
        max  = 1
        color = vvlgreen, vvdgreen

    </plot>

    ## BAT CLUSTERS ##

    <plot>
        show  = yes
        type  = heatmap

        file = ../BAT_regions.txt
        r1  = eval(sprintf("%fr",conf(track_start)-
counter(plot)*(conf(track_width)+conf(track_pad))))
        r0  = eval(sprintf("%fr",conf(track_start)-
counter(plot)*(conf(track_width)+conf(track_pad))-
conf(track_width)))
```

```
        min  = 0
        max  = 1
        color = vvlred, mydred

    </plot>

    ## OMT CLUSTERS ##

    <plot>
        show  = yes
        type  = heatmap

        file = ../OMT_regions.txt
        r1  = eval(sprintf("%fr",conf(track_start)-
counter(plot)*(conf(track_width)+conf(track_pad))))
        r0  = eval(sprintf("%fr",conf(track_start)-
counter(plot)*(conf(track_width)+conf(track_pad))-
conf(track_width)))
        min  = 0
        max  = 1
        color = vvlblue, mydblue

    </plot>

    ## diTPS CLUSTERS ##

    <plot>
        show  = yes
        type  = heatmap

        file = ../diTPS_regions.txt
        r1  = eval(sprintf("%fr",conf(track_start)-
counter(plot)*(conf(track_width)+conf(track_pad))))
        r0  = eval(sprintf("%fr",conf(track_start)-
counter(plot)*(conf(track_width)+conf(track_pad))-
conf(track_width)))
        min  = 0
        max  = 1
        color = vvlpurple, vvdpurple

    </plot>

</plots>

<image>
    <<include etc/image.conf>>
</image>

<colors>
    mygreen = 74,108,1
    mydblue = 7,43,96
    mydred = 82,0,10
```

```
</colors>

<<include etc/colors_fonts_patterns.conf>>

<<include etc/housekeeping.conf>>
```

The karyotype file `karyotype_info.txt` is formatted as below:

```
chr - ptg000010l_1 10 0 10039330 vlpurple
chr - ptg000001l_1 1 0 34834873 vlpurple
chr - ptg000002l_1 2 0 33245114 vlpurple
chr - ptg000003l_1 3 0 72546039 vlpurple
chr - ptg000004l_1 4 0 51580435 vlpurple
chr - ptg000012l_1 12 0 12455358 vlpurple
chr - ptg000005l_1 5 0 24007070 vlpurple
chr - ptg000006l_1 6 0 41401808 vlpurple
chr - ptg000013l_1 13 0 7636326 vlpurple
chr - ptg000015l_1 15 0 16644552 vlpurple
chr - ptg000007l_1 7 0 67192042 vlpurple
chr - ptg000008l_1 8 0 72015906 vlpurple
chr - ptg000009l_1 9 0 36115402 vlpurple
chr - ptg000011l_1 11 0 29022459 vlpurple
chr - ptg000014l_1 14 0 30874415 vlpurple
```

All files used for plotting in CIRCOS have 3 BED-formatted (ie., chromosome-start-end) and a statistic for plotting in the fourth column.

A subset of `gc_content.txt` is shown as below. This contains GC content averaged over 500 kb windows with a 100 kb step size.

```
ptg000001l_1    0     500000     0.364210
ptg000001l_1    100000     600000     0.368040
ptg000001l_1    200000     700000     0.362854
ptg000001l_1    300000     800000     0.367344
ptg000001l_1    400000     900000     0.375780
ptg000001l_1    500000     1000000    0.373644
ptg000001l_1    600000     1100000    0.369934
```

To generate `gc_content.txt` we used the following python functions. Use the assembly as `fasta_file` and assign output to `gc_content.txt`

```
from Bio import SeqIO
from Bio.SeqUtils import GC

def calculate_gc_content(sequence):
    return GC(sequence) / 100
```

```
def generate_table_from_fasta(fasta_file, output_file):
    with open(output_file, "w") as output:
        with open(fasta_file, "r") as handle:
            for record in SeqIO.parse(handle, "fasta"):
                scaffold_name = record.id
                sequence = str(record.seq)
                seq_length = len(sequence)
                window_size = 500000
                overlap = 100000

                for start_pos in range(0, seq_length -
window_size + 1, overlap):
                    end_pos = start_pos + window_size
                    gc_content =
calculate_gc_content(sequence[start_pos:end_pos])

output.write(f"{scaffold_name}\t{start_pos}\t{end_pos}\t{gc_conte
nt:.6f}\n")
                if seq_length % window_size != 0:
                    start_pos = seq_length - window_size
                    end_pos = seq_length
                    gc_content =
calculate_gc_content(sequence[start_pos:end_pos])

output.write(f"{scaffold_name}\t{start_pos}\t{end_pos}\t{gc_conte
nt:.6f}\n")
```

`repeat_content.txt` was similarly generated with the python functions below, using the softmasked genome (from RepeatMasker) as `fasta_file` and `repeat_content.txt` as output.

```
from Bio import SeqIO

# Function to calculate repeat content for a sequence segment
def calculate_repeat_content(sequence):
    total_bases = len(sequence)
    lower_case_bases = sum(1 for base in sequence if
base.islower())
    return lower_case_bases / total_bases if total_bases > 0 else
0

# Function to parse FASTA file and generate table
def generate_rep_table_from_fasta(fasta_file, output_file):
    with open(output_file, "w") as output:
        with open(fasta_file, "r") as handle:
            for record in SeqIO.parse(handle, "fasta"):
                scaffold_name = record.id
                sequence = str(record.seq)
                seq_length = len(sequence)
                window_size = 500000  # Adjust this as needed for
your specific window size
```

```
                overlap = 100000      # Adjust this as needed for
your specific overlap size

                # Process the sequence in windows
                for start_pos in range(0, seq_length -
window_size + 1, overlap):
                    end_pos = start_pos + window_size
                    repeat_content =
calculate_repeat_content(sequence[start_pos:end_pos])

output.write(f"{scaffold_name}\t{start_pos}\t{end_pos}\t{repeat_c
ontent:.6f}\n")
                if seq_length % window_size != 0:
                    start_pos = seq_length - window_size
                    end_pos = seq_length
                    repeat_content =
calculate_repeat_content(sequence[start_pos:end_pos])

output.write(f"{scaffold_name}\t{start_pos}\t{end_pos}\t{repeat_c
ontent:.6f}\n")
```

`gene_counts.txt` was generated using the following python code, and used to plot gene density in 500 kb sliding windows. The code requires chromosome/contig names and lengths, as well as a GTF (general transfer format) file which has been filtered to include only genes `gtf_containing_genes_only.gtf`. Output is `gene_counts.txt`

```
import pandas as pd

# Create a df of chromosome/contig lengths (contig-0-end)
chrom_lengths = {
    'Contig': ['ptg000001l_1', 'ptg000002l_1', 'ptg000003l_1',
'ptg000004l_1', 'ptg000005l_1',
               'ptg000006l_1', 'ptg000007l_1', 'ptg000008l_1',
'ptg000009l_1', 'ptg000010l_1',
               'ptg000011l_1', 'ptg000012l_1', 'ptg000013l_1',
'ptg000014l_1', 'ptg000015l_1'],
    'Start': [0] * 15,  # Adding a third column with zeros for
all rows
    'Stop': [34834873, 33245114, 72546039, 51580435, 24007070,
41401808, 67192042,
             72015906, 36115402, 10039330, 29022459, 12455358,
7636326, 30874415, 16644552]
}

chrom_lengths = pd.DataFrame(chrom_lengths)

# Read in a GTF file containing all genes as a DataFrame
# The GTF file can be filtered previously in bash

genes_gtf = 'gtf_containing_genes_only.gtf'
```

```
columns = ["chromosome", "source", "feature", "start", "end",
"score", "strand", "frame", "attribute"]
df = pd.read_csv(genes_gtf, sep="\t", header=None, names=columns)


# Function for calculating gene density in 500kb windows, and
writing table to used in circos

def calculate_window_counts_and_write(chromosomes_df, output_file):
    with open(output_file, 'w') as f:
        for chromosome_name, chromosome_df in
chromosomes_df.groupby("chromosome"):
            window_start = 0
            window_end = 500000
            step_size = 100000

            while window_end <= chromosome_df["end"].max():
                count = ((chromosome_df["start"] >= window_start) &
(chromosome_df["end"] <= window_end)).sum()
                line =
f"{chromosome_name}\t{window_start}\t{window_end}\t{count}\n"
                f.write(line)
                window_start += step_size
                window_end += step_size
```

To plot clusters of genes encoding specific enzyme classes (inner rings), we generated files in where regions between the start and end of a gene were assigned 1 and all other regions of the genome were assigned 0. As an example, here are some BAHD acyl transferase (BAT) positions in chromosome 1 and 2:

```
ptg000001l_1    0     34834873   0 # no BAT in contig 1
ptg000002l_1    0     3277664    0
ptg000002l_1    3277664    3280436    1 # BAT
ptg000002l_1    3280436    3287913    0
ptg000002l_1    3287913    3290129    1 # BAT
ptg000002l_1    3290129    3314777    0
ptg000002l_1    3314777    3317032    1 # BAT
ptg000002l_1    3317032    4269566    0
ptg000002l_1    4269566    4270951    1 # BAT
ptg000002l_1    4270951    4506871    0
```

To generate gene region files (`CYP_regions.txt, BAT_regions.txt, OMT_regions.txt, diTPS_regions.txt`), we first obtained GTF files that were filtered to include only genes corresponding to each of the target classes. These were used in the following python code (using `BAT_genes.gtf to` generate `BAT_regions.txt` in this example):

```
import pandas as pd

# data frame of chromosome/contig lengths, as above
chrom_lengths = {
```

```
    'Contig': ['ptg000001l_1', 'ptg000002l_1', 'ptg000003l_1',
'ptg000004l_1', 'ptg000005l_1',
               'ptg000006l_1', 'ptg000007l_1', 'ptg000008l_1',
'ptg000009l_1', 'ptg000010l_1',
               'ptg000011l_1', 'ptg000012l_1', 'ptg000013l_1',
'ptg000014l_1', 'ptg000015l_1'],
    'Start': [0] * 15,
    'Stop': [34834873, 33245114, 72546039, 51580435, 24007070,
41401808, 67192042,
             72015906, 36115402, 10039330, 29022459, 12455358,
7636326, 30874415, 16644552]
}

chrom_lengths = pd.DataFrame(chrom_lengths)

# Read the BAT GTF file into a DataFrame
columns = ["chromosome", "source", "feature", "start", "end",
"score", "strand", "frame", "attribute"]
df_BATS = pd.read_csv("BAT_genes.gtf", sep="\t", header=None,
names=columns)

# Initialize df for output
complete_df = pd.DataFrame(columns=['chromosome', 'start', 'end',
'indicator'])

for _, row in chrom_lengths.iterrows():
    # Initialize the start of the chromosome
    current_start = row['Start']
    # Extract all genes within this chromosome
    genes = df_BATS[df_BATS['chromosome'] == row['Contig']]

    # Initialize a list to hold all dfs for concatenation
    temp_dfs = []

    # Process each gene
    for _, gene in genes.iterrows():
    # Check for a gap between the current start and the gene
start
        if gene['start'] > current_start:
            # There is a gap; create a df for it, assigned 0
            temp_dfs.append(pd.DataFrame({
                'chromosome': [row['Contig']],
                'start': [current_start],
                'end': [gene['start']],
                'indicator': [0]
            }))

        # Add the gene region
        temp_dfs.append(pd.DataFrame({
            'chromosome': [row['Contig']],
            'start': [gene['start']],
            'end': [gene['end']],
```

```
            'indicator': [1]
        }))

        # Update the current start to the end of the current gene
        current_start = gene['end']

    # Check if there is remaining distance to cover after the
last gene
    if current_start < row['Stop']:
        # Add the remaining region with indicator 0
        temp_dfs.append(pd.DataFrame({
            'chromosome': [row['Contig']],
            'start': [current_start],
            'end': [row['Stop']],
            'indicator': [0]
        }))

    # Concatenate all df parts for this chromosome into the main
df
    if temp_dfs:
        complete_df = pd.concat([complete_df, pd.concat(temp_dfs,
ignore_index=True)], ignore_index=True)

# Convert 'start', 'end', and 'indicator' to integers
complete_df[['start', 'end', 'indicator']] =
complete_df[['start', 'end', 'indicator']].astype(int)

# Save to file
complete_df.to_csv('BAT_regions.txt', sep='\t', index=False,
header=False)
```
