# Supplementary material for: A chromosome level reference genome of Diviner’s sage (Salvia divinorum) provides insight into salvinorin A biosynthesis
Source: BMC Plant Biol. 2024 Oct 1;24:914. doi: 10.1186/s12870-024-05633-0 (PMC11443658; doi:10.1186/s12870-024-05633-0)
Supplement: Supplementary file 5 — Supplementary Material 5. [file 12870_2024_5633_MOESM5_ESM.pdf]

Supplementary Methods file 2

A chromosome level reference genome of Diviner's sage (*Salvia divinorum*) provides insight into salvinorin A biosynthesis

Scott A. Ford, Rob W. Ness, Moonhyuk Kwon, Dae-Kyun Ro, Michael A. Phillips

Analysis of RNA-seq Data

Contents

Mapping MeJA-induced and control reads……………….2
Analyzing MeJA-induced expression………………………5
Mapping trichome reads ………………………………….....7
Analyzing trichome expression……………………………..9

Trimmomatic

In [ ]:
```
ls ../../raw_data/JasmonateTreatmentRNASeq/
```

In [ ]:
```
import os, glob,subprocess
input_data = {}
for f in glob.glob("../../raw_data/JasmonateTreatmentRNASeq/*.fastq"):
    prefix = f.split(".")[-2][:-3]
    if prefix not in input_data:input_data[prefix] = {"left":None, "right":N
    if "R1" in f:input_data[prefix]['left']=f
    elif "R2" in f:input_data[prefix]['right']=f

input_data
```

In [ ]:
```
#!mkdir -p ../../data/Jasmonate_RNASeq_trimmed/
```

In [ ]:
```
fastq_path = "../../raw_data/JasmonateTreatmentRNASeq/"
output_path = "../../data/Jasmonate_RNASeq_trimmed/"
head = "HI.5205.008.Index_"
for prefix in input_data:
    in1_fq = input_data[prefix]['left']
    in2_fq = input_data[prefix]['right']
    outpaired1_fq   = "{output_path}{prefix}.trim.paired.1.fq.gz".format(out
    outUNpaired1_fq = "{output_path}{prefix}.trim.UNpaired.1.fq.gz".format(o
    outpaired2_fq   = "{output_path}{prefix}.trim.paired.2.fq.gz".format(out
    outUNpaired2_fq = "{output_path}{prefix}.trim.UNpaired.2.fq.gz".format(o
    cmd ="""java -jar /research/tmp_apps/Trimmomatic-0.36/trimmomatic-0.36.j
PE \
-threads 2 \
-phred33 \
{in_fwd} {in_rev} \
{outpaired1} \
{outUNpaired1} \
{outpaired2} \
{outUNpaired2} \
ILLUMINACLIP:/research/tmp_apps/Trimmomatic-0.36/adapters/TruSeq3-PE.fa:2:30
LEADING:3 \
TRAILING:3 \
SLIDINGWINDOW:4:15 \
MINLEN:36""".format(in_fwd=in1_fq,in_rev=in2_fq,outpaired1=outpaired1_fq, ou
    print(cmd, "\n")
    p = subprocess.Popen(cmd, shell=True)
    #print(p.communicate()[-1])
```

Organize genome files:

Draft genome copied from here

/research/projects/chlamydomonas/lipid_BulkSegregant/Pacbio/analysis/working_assemblie

Draft Annotation copied from here

/research/projects/chlamydomonas/lipid_BulkSegregant/Pacbio/analysis/BRAKER_test/Sd_ar

In [ ]:
```
!cp /research/projects/chlamydomonas/lipid_BulkSegregant/Pacbio/analysis/wor
```

Build STAR index

In [ ]:
```
%%bash

STAR \
--runThreadN 24 \
    --runMode genomeGenerate \
    --genomeDir ../../data/Sdiv \
    --genomeFastaFiles ../../raw_data/draftGenome/S_divinorum_filtered_lengt
    --sjdbGTFfile      ../../raw_data/draftGenome/S_divinorum_filtered_lengt
    --sjdbOverhang 99 \
    --genomeSAindexNbases 13
```

STAR Alignment

In [ ]:
```
%%bash

parallel -j 8 \
"STAR --runThreadN 6 \
--readFilesIn ../../data/Jasmonate_RNASeq_trimmed/{}.trim.paired.1.fq \
              ../../data/Jasmonate_RNASeq_trimmed/{}.trim.paired.2.fq \
--genomeDir ../../data/Sdiv \
--outSAMstrandField intronMotif \
--outSAMtype BAM SortedByCoordinate \
--outFileNamePrefix ../../data/mapping/{}/{}- " ::: \
SDL_Control_1 \
SDL_Control_2 \
SDL_Control_3 \
SDL_Control_4 \
```

```
SDL_MeJA_1 \
SDL_MeJA_2 \
SDL_MeJA_3 \
SDL_MeJA_4
```

AddOrReplaceReadGroups

In [ ]:
```
#!mkdir ../../data/BAMs/
```

In [ ]:
```
%%bash

parallel -j 8 \
"gatk AddOrReplaceReadGroups \
-I ../../data/mapping/{}/{}-Aligned.sortedByCoord.out.bam \
-O ../../data/BAMs/{}.RG.bam \
--RGID {} \
--RGLB lib1 \
--RGPL illumina \
--RGPU unit1 \
--RGSM {} \
--VALIDATION_STRINGENCY LENIENT" ::: \
SDL_Control_1 \
SDL_Control_2 \
SDL_Control_3 \
SDL_Control_4 \
SDL_MeJA_1 \
SDL_MeJA_2 \
SDL_MeJA_3 \
SDL_MeJA_4
```

Index BAMs

In [ ]:
```
!parallel -j 8 "samtools index {}" ::: ../../data/BAMs/*bam
```

In [ ]:
```
print("done")
```

In [ ]:
```
library("Rsamtools")
```

Assemble information.

- include BAMs from Mapping.*.RNASeq.ipynb

In [ ]:
```
# find your files
sampleTable <- read.table('sample_data.txt', header = T)
```

In [ ]:
```
filenames <- file.path("../../data/BAMs", paste0(sampleTable$sample_ID, ".RG
file.exists(filenames)
```

In [ ]:
```
bamfiles <- BamFileList(filenames, yieldSize=2000000)
```

Annotate genes from de novo gene models

In [ ]:
```
library("GenomicFeatures")
```

In [ ]:
```
gtffile <- file.path("../../raw_data/draftGenome//S_divinorum_filtered_lengt
file.exists(gtffile)
```

In [ ]:
```
txdb <- makeTxDbFromGFF(gtffile, format = "gff3", circ_seqs = character())
```

In [ ]:
```
ebg <- exonsBy(txdb, by="gene")
```

Summarize read overlaps on genes

In [ ]:
```
library("GenomicAlignments")
library("BiocParallel")
```

In [ ]:
```
se <- summarizeOverlaps(features=ebg, reads=bamfiles,
                        mode="Union",
                        singleEnd=FALSE,
                        ignore.strand=TRUE,
                        fragments=TRUE )
```

In [ ]:
```
save(se, file = "full_SE.RData")
```

In [ ]:
```
colData(se) <- DataFrame(sampleTable)
```

In [ ]:
```
se$treatment
```

Analyse differential selection between control and jasmonate treatments

In [ ]:
```
library("DESeq2")
```

In [ ]:
```
dds <- DESeqDataSet(se, design = ~ treatment)
```

In [ ]:
```
dds <- DESeq(dds)
res <- results(dds)
```

In [ ]:
```
res_df <- data.frame(res)
```

Write differential expression to file

In [ ]:
```
write.table(res_df, file="results.txt", sep="\t")
```

Mapping Trichome Reads

Trimmomatic to trim Reads

In [ ]:
```
%%bash

java -jar /research/tmp_apps/Trimmomatic-0.36/trimmomatic-0.36.jar PE \
-threads 24 -phred33 \
../../raw_data/trichomeRNA/SRR3716680_1.fastq.gz \
../../raw_data/trichomeRNA/SRR3716680_2.fastq.gz \
../../data/Jasmonate_RNASeq_trimmed/SRR3716680.trim.paired.1.fq.gz \
../../data/Jasmonate_RNASeq_trimmed/SRR3716680.trim.UNpaired.1.fq.gz \
../../data/Jasmonate_RNASeq_trimmed/SRR3716680.trim.paired.2.fq.gz \
../../data/Jasmonate_RNASeq_trimmed/SRR3716680.trim.UNpaired.2.fq.gz \
ILLUMINACLIP:/research/tmp_apps/Trimmomatic-0.36/adapters/TruSeq3-PE.fa:2:30
```

In [ ]:
```
!mkdir ../../data/mapping/Trichome
```

Unzip Reads

In [ ]:
```
!gunzip ../../data/Jasmonate_RNASeq_trimmed/SRR3716680.trim.paired.1.fq.gz
!gunzip ../../data/Jasmonate_RNASeq_trimmed/SRR3716680.trim.paired.2.fq.gz
```

Align reads to S. divinorum assembly using STAR

In [ ]:
```
%%bash
STAR --runThreadN 6 \
--readFilesIn ../../data/Jasmonate_RNASeq_trimmed/SRR3716680.trim.paired.1.f
              ../../data/Jasmonate_RNASeq_trimmed/SRR3716680.trim.paired.2.f
--genomeDir ../../data/Sdiv \
--outSAMstrandField intronMotif \
--outSAMtype BAM SortedByCoordinate \
--outFileNamePrefix ../../data/mapping/Trichome/SRR3716680
```

Add Read Group Labels

In [ ]:
```
%%bash
parallel -j 8 \
```

```
"gatk AddOrReplaceReadGroups \
-I ../../data/mapping/Trichome/{}Aligned.sortedByCoord.out.bam \
-O ../../data/BAMs/{}.RG.bam \
--RGID {} \
--RGLB lib1 \
--RGPL illumina \
--RGPU unit1 \
--RGSM {} \
--VALIDATION_STRINGENCY LENIENT" ::: \
SRR3716680
```

In [ ]:

```

```

In [ ]:

```
!samtools index ../../data/BAMs/SRR3716680.RG.bam
```

In [ ]:

```

```

In [ ]:
```
library("Rsamtools")
```

Assemble information.

- include BAMs from Mapping.Trichome.RNASeq.ipynb and Mapping.Jasmonate.RNASeq.ipynb

In [ ]:
```
# find your files
sampleTable <- read.table('sample_data.wTrichome.txt', header = T)
```

In [ ]:
```
filenames <- file.path("../../data/BAMs", paste0(sampleTable$sample_ID, ".RG
```

First step

In [ ]:
```
bamfiles <- BamFileList(filenames, yieldSize=2000000)
```

Annotate genes from de novo gene models

In [ ]:
```
library("GenomicFeatures")
```

In [ ]:
```
gtffile <- file.path("../../raw_data/draftGenome//S_divinorum_filtered_lengt
```

In [ ]:
```
txdb <- makeTxDbFromGFF(gtffile, format = "gff3", circ_seqs = character())
```

In [ ]:
```
ebg <- exonsBy(txdb, by="gene")
```

Summarize read overlaps on genes

In [ ]:
```
library("GenomicAlignments")
library("BiocParallel")
```

In [ ]:
```
se <- summarizeOverlaps(features=ebg, reads=bamfiles,
                        mode="Union",
                        singleEnd=FALSE,
                        ignore.strand=TRUE,
                        fragments=TRUE )
```

In [ ]:
```
save(se, file = "full_SE.wTrichomes.RData")
```

In [ ]:
```
write.table(assay(se), "tmp.se.txt")
```

```
In [ ]: colData(se) <- DataFrame(sampleTable)
```

```
In [ ]: se$treatment
```

Analyze fold change without replication following exploratory guidelines in EdgeR

```
In [ ]: library(edgeR)
```

```
In [ ]: names(colData(se)) = "group"
        dge = DGEList(se)
```

```
In [ ]: x <- read.delim("tmp.se.txt", sep=' ')
```

```
In [ ]: x_pared <- x[,c(1,2,3,4,9)]
```

```
In [ ]: group <- factor(c(1,1,1,1,2))
```

```
In [ ]: y_pared <- DGEList(counts=x_pared,group=group)
```

```
In [ ]: keep <- filterByExpr(y_pared)
        y_pared <- y_pared[keep,,keep.lib.sizes=FALSE]
        y_pared <- normLibSizes(y_pared)
        head(y_pared)
```

```
In [ ]: y_pared$S_divinorum_g1000
```

```
In [ ]: #estimate dispersion from control reps
        y1 <- y_pared
        y1$samples$group <- 1
        y0 <- estimateDisp(y1, trend="none", tagwise=FALSE)
        bcv <- y0$common.dispersion
        et <- exactTest(y_pared, dispersion=bcv^2)
```

Write fold change of trichome specific sequence vs leaf

```
In [ ]: write.table(et, 'foldchange.trichome_vs_leaf.txt', sep="\t")
```
